# Longitudinal monitoring of cell-free DNA methylation in *ALK*-positive non-small cell lung cancer patients

**DOI:** 10.1186/s13148-022-01387-4

**Published:** 2022-12-02

**Authors:** Florian Janke, Arlou Kristina Angeles, Anja Lisa Riediger, Simone Bauer, Martin Reck, Albrecht Stenzinger, Marc A. Schneider, Thomas Muley, Michael Thomas, Petros Christopoulos, Holger Sültmann

**Affiliations:** 1 Division of Cancer Genome Research, German Cancer Research Center, National Center for Tumor Diseases, Heidelberg, Germany; 2 German Center for Lung Research (DZL), TLRC Heidelberg, Heidelberg, Germany; 3 Helmholtz Young Investigator Group, Multiparametric Methods for Early Detection of Prostate Cancer, German Cancer Research Center (DKFZ), Heidelberg, Germany; 4 Department of Urology, Heidelberg University Hospital, Heidelberg, Germany; 5 Faculty of Biosciences, Heidelberg University, Heidelberg, Germany; 6 Lung Clinic Grosshansdorf, Airway Research Center North, German Center for Lung Research, Grosshansdorf, Germany; 7 Institute of Pathology, Heidelberg University Hospital, Heidelberg, Germany; 8 German Cancer Consortium (DKTK), Heidelberg, Germany; 9 Translational Research Unit, Thoraxklinik at University Hospital Heidelberg, Heidelberg, Germany; 10 Department of Oncology, Thoraxklinik and National Center for Tumor Disease (NCT) at Heidelberg University Hospital, Heidelberg, Germany

**Keywords:** *ALK*-positive NSCLC, Biomarkers, Cell-free DNA, cfMeDIP-seq, DNA methylation, Liquid biopsy, Epigenetics

## Abstract

**Background:**

DNA methylation (5-mC) signals in cell-free DNA (cfDNA) of cancer patients represent promising biomarkers for minimally invasive tumor detection. The high abundance of cancer-associated 5-mC alterations permits parallel and highly sensitive assessment of multiple 5-mC biomarkers. Here, we performed genome-wide 5-mC profiling in the plasma of metastatic *ALK*-rearranged non-small cell lung cancer (NSCLC) patients receiving tyrosine kinase inhibitor therapy. We established a strategy to identify *ALK*-specific 5-mC changes from cfDNA and demonstrated the suitability of the identified markers for cancer detection, prognosis, and therapy monitoring.

**Methods:**

Longitudinal plasma samples (*n* = 79) of 21 *ALK*-positive NSCLC patients and 13 healthy donors were collected alongside 15 *ALK*-positive tumor tissue and 10 healthy lung tissue specimens. All plasma and tissue samples were analyzed by cell-free DNA methylation immunoprecipitation sequencing to generate genome-wide 5-mC profiles. Information on genomic alterations (i.e., somatic mutations/fusions and copy number alterations) determined in matched plasma samples was available from previous studies.

**Results:**

We devised a strategy that identified tumor-specific 5-mC biomarkers by reducing 5-mC background signals derived from hematopoietic cells. This was followed by differential methylation analysis (cases vs. controls) and biomarker validation using 5-mC profiles of *ALK*-positive tumor tissues. The resulting 245 differentially methylated regions were enriched for lung adenocarcinoma-specific 5-mC patterns in TCGA data and indicated transcriptional repression of several genes described to be silenced in NSCLC (e.g., *PCDH10*, *TBX2*, *CDO1*, and *HOXA9*). Additionally, 5-mC-based tumor DNA (5-mC score) was highly correlated with other genomic alterations in cell-free DNA (Spearman, *ρ* > 0.6), while samples with high 5-mC scores showed significantly shorter overall survival (log-rank *p* = 0.025). Longitudinal 5-mC scores reflected radiologic disease assessments and were significantly elevated at disease progression compared to the therapy start (*p* = 0.0023). In 7 out of 8 instances, rising 5-mC scores preceded imaging-based evaluation of disease progression.

**Conclusion:**

We demonstrated a strategy to identify 5-mC biomarkers from the plasma of cancer patients and integrated them into a quantitative measure of cancer-associated 5-mC alterations. Using longitudinal plasma samples of ALK-positive NSCLC patients, we highlighted the suitability of cfDNA methylation for prognosis and therapy monitoring.

**Supplementary Information:**

The online version contains supplementary material available at 10.1186/s13148-022-01387-4.

## Background

Liquid biopsies from circulating cell-free DNA (cfDNA) have demonstrated their utility for minimally invasive cancer detection, tumor genotyping, resistance as well as residual disease monitoring during therapy [1–7]. The analysis of cfDNA molecules carrying genomic aberrations (i.e., somatic mutations or copy number alterations [CNAs]) is highly tumor-specific and allows accurate detection and longitudinal assessment of cancers. However, this approach poses several challenges, including the low abundance of mutated cfDNA fragments, a lack of common mutations across patient groups and the necessity to have a priori knowledge of a tumor’s molecular profile [3, 8]. DNA methylation, occurring at the 5’-carbon of cytosines (5-mC), was demonstrated to be preserved in cfDNA [9–11] and represents a biomarker with the potential to overcome some of these limitations. Aberrant methylation at cytosine–guanine dinucleotides (CpGs) is central to carcinogenesis and usually occurs genome-wide [12–14]. This allows parallel assessment of multiple 5-mC sites, thereby increasing the probability to capture cancer-derived signals in the circulation. In addition, tumors without known genomic alterations (i.e., mutations or CNAs) may be detected utilizing cancer-specific 5-mC signatures. With its gene regulatory function, 5-mC contains additional information about the tumor that cannot be derived from genomic cfDNA alterations. Its presence at regulatory regions, such as promoters or enhancers, represses transcription of associated genes. Previous reports were able to deduce silenced tumor suppressors [15] and cell type-specific gene regulation (i.e., tumor localization) from cell-free 5-mC profiles [9, 11, 16–19]. Cell-free methylation DNA immunoprecipitation followed by high-throughput sequencing (cfMeDIP-seq) is a sensitive approach to detect 5-mC signals from low amounts of DNA (> 1 ng). The enrichment of methylated cfDNA molecules allows genome-scale 5-mC profiling without error-prone bisulfite conversion [10, 20]. In principle, cfMeDIP-seq enables concurrent assessment of numerous 5-mC tumor biomarkers [10, 21–23]. Yet, their identification in cfDNA presents a challenge, because cfDNA is regarded as a mixture of DNA fragments released from various cell and tissue types. Most cfDNA is derived from hematopoietic cells, while tumor-derived DNA molecules commonly compose a minor fraction (< 1%) [11, 18, 19]. This poses the difficulty in identifying tumor-informative 5-mC signals within the vast amount of non-cancer background DNA.

So far, few studies addressed the capability of cfMeDIP-seq for the assessment of longitudinal therapy kinetics [21, 24, 25]. Here, we applied cfMeDIP-seq to longitudinally sampled plasma of non-small cell lung cancer (NSCLC) patients with oncogenic rearrangements of the anaplastic lymphoma kinase (*ALK*) gene. These patients are susceptible to *ALK* tyrosine kinase inhibitor (TKI) therapy and can experience long survival under serial treatment with multiple targeted drugs [26–28]. However, therapy failure due to acquired drug resistance is common [27]. Therefore, timely recognition of disease progression and consequential adaptation of therapy strategy is desirable for better disease management. We implemented a strategy to identify tumor-specific 5-mC biomarkers from cfMeDIP-seq data of cancer patient cfDNA. Our approach uses public whole-genome bisulfite sequencing (WGBS) datasets of cell types composing the non-tumor fraction of cfDNA [18] to identify and reduce confounding 5-mC background signals. We validated the tumor specificity of the resulting 5-mC biomarkers using lung cancer tissue methylation and gene expression data, systematically compared them to genomic alterations previously determined in matched plasma [2, 4], and followed their abundances in serial plasma samples taken during TKI therapy. The results of this study highlight the complementarity of epigenomic and genomic cfDNA analysis and, for the first time, demonstrate the applicability of cfMeDIP-seq for longitudinal treatment monitoring in *ALK*-positive NSCLC. Additionally, we provide a strategy for 5-mC biomarker identification that can be applied in future studies.

## Results

### Patient characteristics

A total of 66 plasma specimens from 21 metastatic *ALK*-positive NSCLC lung adenocarcinoma (LUAD) patients were included in this study (Table 1). Longitudinal plasma was available for eleven patients ranging from 2 to 14 consecutive samples. Baseline tissue biopsies identified *EML4-ALK* fusion variant 1 (V1; E13:A20) in 43% (9/21), V2 (E20:A20) in 10% (2/21), V3 (E6:A20) in 33% (7/21), and other variants in 10% (2/21) of patients. *TP53* mutations were detected in baseline tissue biopsies of 29% (6/21) of cases. All patients received one or multiple lines of *ALK*-directed TKI therapy, and 30 plasma samples were taken at time points of disease recurrence. At the last follow-up date, 10/21 patients had deceased (Additional file 1: Figure S1). Information on genomic alterations of all plasma samples was available from previously published work [2, 4]. This included cell-free abundances of the *EML4-ALK* fusion gene and somatic mutations, determined by hybrid-capture-based sequencing as well as genome-scale chromosomal instability assessment by shallow whole-genome sequencing (sWGS), summarized as trimmed median absolute deviation from copy number neutrality (t-MAD) scores [29].

**Table 1:** Patient characteristics

*ALK*-positive NSCLC patients (*n* = 21; *n* = 66 plasma specimens)	
Age, median (range)	56 (42–80)
Sex, male	11/21
Stage IV	21/21
*ALK fusion variant, patient number*^1^	
EML4-ALK V1/V2	11
EML4-ALK V3	7
Other^2^	2
No data	1
*TP53 status, patient numbe*r^1^	
Positive	6
Negative	14
No data	1
*Treatment, sample numbe*r	
Crizotinib	19
Ceritinib, Alectinib, Brigatinib	27
Lorlatinib	5
Chemotherapy	10
Naïve	4
No data	1
Number of samples per patient, mean (range)	3.1 (1–14)
*Radiological evaluation at sampling, number of samples*	
Extracranial PD	27
Intracranial PD	4
SD	30
PR	2
No data	3

*ALK* anaplastic lymphoma kinase, *EML4* echinoderm microtubule-associated protein-like 4, *KCL1* kinesin light chain 1, *NGS* next-generation sequencing, *PD* progressive disease, *PR* partial response, *SD* stable disease, *TP53* tumor protein 53

^1^Data available for 20/21 patients from NGS of tissue biopsies at diagnosis of stage IV disease

^2^One patient with a K9A20 (*KCL1*) and one with an E9A10 fusion

### Identification of tumor-informative cell-free 5-mC biomarkers

**Fig. 1:**
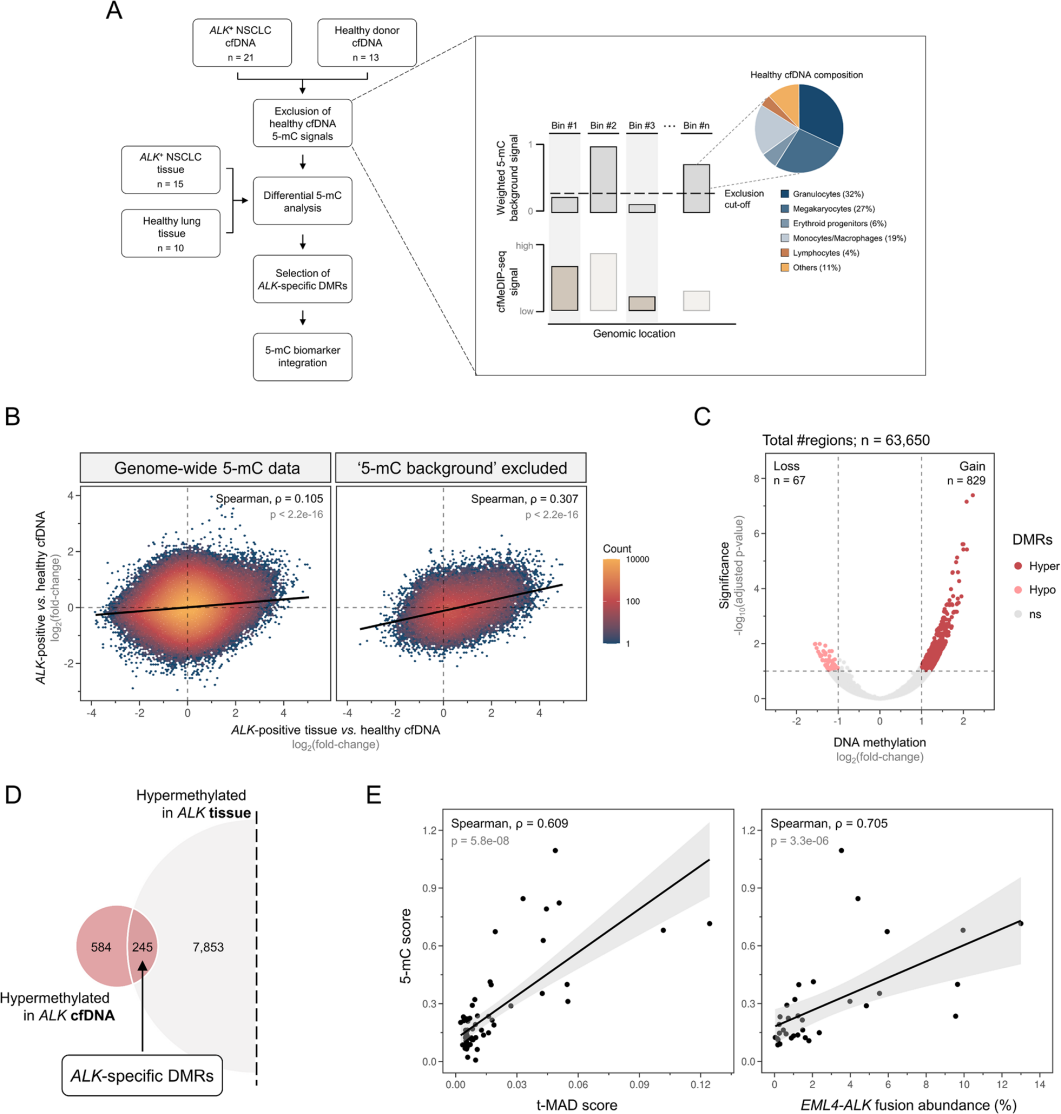
Identification of tumor-informative 5-mC regions from ALK-positive cfDNA. **A** 5-mC biomarker identification workflow overview. **B** Density plot illustrating log_2_ 5-mC differences between *ALK*-positive patient and healthy control cfDNA (y-axis) against *ALK* patient tissue *versus* healthy control cfDNA (*x*-axis). Healthy control cfDNA samples used as reference for log_2_(fold-change) calculation were split, using one half as reference for *ALK* cfDNA (*n* = 6) and the other for *ALK* tissue samples (*n* = 6). The left plot shows the correlation at all evaluable genomic regions (*n* = 2,596,067). The right one shows the correlation after exclusion of ‘5-mC background ‘signals (*n* = 63,650). **C** Volcano plot of 63,650 genomic regions remaining after ‘5-mC background ‘exclusion. Significantly hyper- and hypomethylated sites, comparing 21 *ALK*-positive to 13 healthy control cfDNA samples, are highlighted in dark and light red, respectively. **D** Overlap of *ALK* cfDNA hyper-DMRs and hyper-DMRs detected in *ALK* tissue. **E** Scatter plots showing the correlation between 5-mC scores calculated from 245 *ALK*-specific hyper-DMRs and t-MAD scores (left) and *EML4-ALK* fusion abundances (right). Points represent individual samples and black line denotes fitted linear regression model with 95% confidence interval. *ALK*, anaplastic lymphoma kinase; cfDNA, cell-free DNA; DMR, differentially methylated region; *EML4*, echinoderm microtubule-associated protein-like 4; ns, not significant; NSCLC, non-small cell lung cancer; t-MAD, trimmed median absolute deviation from copy number neutrality

To identify tumor-associated 5-mC biomarkers in cfDNA, we first generated genome-wide DNA methylation profiles from 66 *ALK*-positive and 13 healthy control plasma samples by cfMeDIP-seq. We chose 21 patient samples for the biomarker identification process by selecting one sample per individual in cases with available serial plasma. Hereby, longitudinal samples with detectable genomic alterations (previously measured in the same plasma [2, 4]) were favored, reasoning that these samples were expected to contain elevated amounts of tumor-derived cfDNA and were therefore well suited for biomarker identification (selected samples are specified in Additional file 1: Table S1). In addition to plasma, *ALK*-positive tumor (*n* = 15) and normal lung tissue (*n* = 10) samples were subjected to cfMeDIP-seq. The marker identification strategy established in this study interrogated 5-mC coverage profiles at 7,264,350 non-overlapping genomic windows [18] and selected tumor-informative regions by excluding non-tumor 5-mC signals. This was followed by differential methylation analysis between cases and controls and biomarker validation in *ALK*-positive tumor tissues (Fig. 1A and Additional file 1: Fig. S2).

As most cfDNA molecules originate from various hematopoietic cells [11, 18, 19], we reasoned that frequently methylated genomic regions in these cells contain confounding background 5-mC signals rather than carrying information about a patient’s tumor. To test this, we used publicly available WGBS data of cell types described to compose cfDNA in healthy individuals (i.e., granulocytes, megakaryocytes, erythroid progenitors, monocytes, macrophages, lymphocytes, and other non-hematopoietic cells) and combined them according to their relative contribution to cfDNA (Fig. 1A; Methods) [18]. We found that focusing on genomic regions commonly unmethylated in the combined ‘5-mC background’ (i.e., methylated in the combination of hematopoietic cells) increased the correlation between *ALK*-positive cfDNA and *ALK* tissue 5-mC signals compared to all evaluable regions. The highest correlation was observed at an exclusion threshold of *β* < 0.15 (Fig. 1B; Spearman, *ρ* = 0.307), indicating an enhanced tumor association of the 5-mC signals at the remaining 63,650 sites. Next, we identified cancer-derived differentially methylated regions (DMRs) from cfDNA at ‘5-mC background’-depleted sites by comparing *ALK*-positive patients to healthy controls. 829 hyper- and 67 hypomethylated DMRs were detected in *ALK*-positive cfDNA (Fig. 1C). To validate their tumor association, we overlapped the cfDNA-derived DMRs to differentially methylated sites found by comparing *ALK*-positive tumor tissue to normal lung tissue (Additional file 1: Figure S3A). Two hundred and forty-five of 829 (29.6%) DMRs were concordantly hypermethylated in *ALK*-positive cfDNA and tumor tissue samples (Fig. 1D), and the significance of overlaps was confirmed by permutation testing (*p* < 0.0001). Hypomethylated DMRs in cfDNA did not overlap with tumor tissue DNA hypomethylation, most likely due to the limitation of cfMeDIP-seq to detect hypomethylated regions in cfDNA samples. Importantly, we found that differential methylation analysis without prior ‘5-mC background’ depletion identified fewer DMRs whose methylation status could be confirmed in tumor tissue (65/3,948 [1.6%] hyper-DMRs). This suggested that the ‘5-mC background’ depletion facilitates the detection of cancer-derived DMRs. We then developed a metric, termed ‘5-mC score,’ to quantitatively assess the extent of cancer-derived 5-mC changes in each cfDNA sample. The 5-mC score combined the 5-mC signal at the 245 hyper-DMRs with confirmed tumor tissue association by calculating their absolute median coverage deviation from a healthy cfDNA control panel (Methods). A high concordance between 5-mC scores and chromosomal instability (t-MAD score; Spearman, *ρ* = 0.609) as well as 5-mC scores and *EML4-ALK* fusion abundances (Spearman, *ρ* = 0.705) was observed, suggesting that cfMeDIP-seq profiles can inform about the abundance of tumor-derived cfDNA molecules in plasma samples (Fig. 1E). Similar correlations with the t-MAD score and the *EML4-ALK* fusion abundance were found when all 829 hyper-DMRs were considered for the calculation of the 5-mC score (Additional file 1: Figure S3B; Spearman, *ρ* = 0.537 [t-MAD] and *ρ* = 0.657 [*EML4-ALK* fusion]). This suggested that the presented strategy allows the identification of 5-mC biomarkers from cfDNA alone, without additional information from tumor tissue.

### Cell-free 5-mC markers are enriched for lung adenocarcinoma-specific methylation and inform about tissue-specific gene expression

DNA methylation occurs in a tissue-specific manner and plays a part in transcriptional regulation [12]. We used publicly available reference datasets (i.e., Illumina 450 k methylation array and RNA-seq data) from The Cancer Genome Atlas (TCGA) [30, 31] to investigate whether the identified 245 *ALK*-specific hyper-DMRs were informative on LUAD biology. In total, 189 of the 245 hyper-DMRs were covered by at least one cytosine probed by the Illumina 450 k methylation array and 78/189 (41.3%) were concordantly hypermethylated in LUAD (*n* = 455) *versus* adjacent normal lung tissue (*n* = 75). Permutation testing confirmed significant enrichment of LUAD-specific hypermethylation within the hyper-DMRs identified from *ALK*-positive cfDNA (*p* < 0.0001). Interestingly, we observed a similar number of overlapping hyper-DMRs when TCGA-LUAD samples were stratified by pathologic stage or molecular driver (i.e., *EGFR*, *KRAS*, and *EML4-ALK*; Additional file 1: Table S2). This suggested that some of the hyper-DMRs found in cfDNA might be informative of localized cancer in LUAD patients independent of the *ALK*-positive subtype addressed in this study. We next examined whether the identified hyper-DMRs were indicative of the transcriptional status of proximal genes. Thirty-five out of 189 (18.5%) hyper-DMRs, corresponding to 31/135 (23.0%) genes, demonstrated a significant inverse correlation between DNA methylation and gene expression in non-cancer TCGA tissue samples (*n* = 150), suggesting their 5-mC-dependent transcriptional repression. The majority of genomic regions with gene regulatory 5-mC signals resided in CpG islands (31/35) and many were located proximal to promoters (24/35; i.e.,  ≤ 5 kb upstream of the transcription start site and 5’-untranslated regions). Among the associated genes, 23/31 (74.2%) were transcriptionally downregulated in TCGA-LUAD (n = 507) *versus* normal lung tissues (*n* = 288) obtained from the Genotype-Tissue Expression project (GTEx; Additional file 1: Table S3). Promoter hypermethylation and/or transcriptional silencing in NSCLC was previously reported for some of these genes (e.g., *PCDH10*, *TBX2*, *CDO1*, and *HOXA9*). Interestingly, *PCDH10*, *TBX2*, and *CDO1* were described as potential biomarkers for early-stage lung cancers [32–34]. *PCDH10* hypermethylation was associated with adverse disease outcomes after surgery of stage I NSCLC [33], while *TBX2* expression was demonstrated to progressively decrease across premalignant lesions with respect to normal lungs [34]. Of note, promoter 5-mC levels of *CDO1* and *HOXA9* were previously utilized for plasma-based disease assessment in both early and advanced lung cancers [32, 35].

### 5-mC profiling of cfDNA complements CNA and mutation analysis

**Fig. 2:**
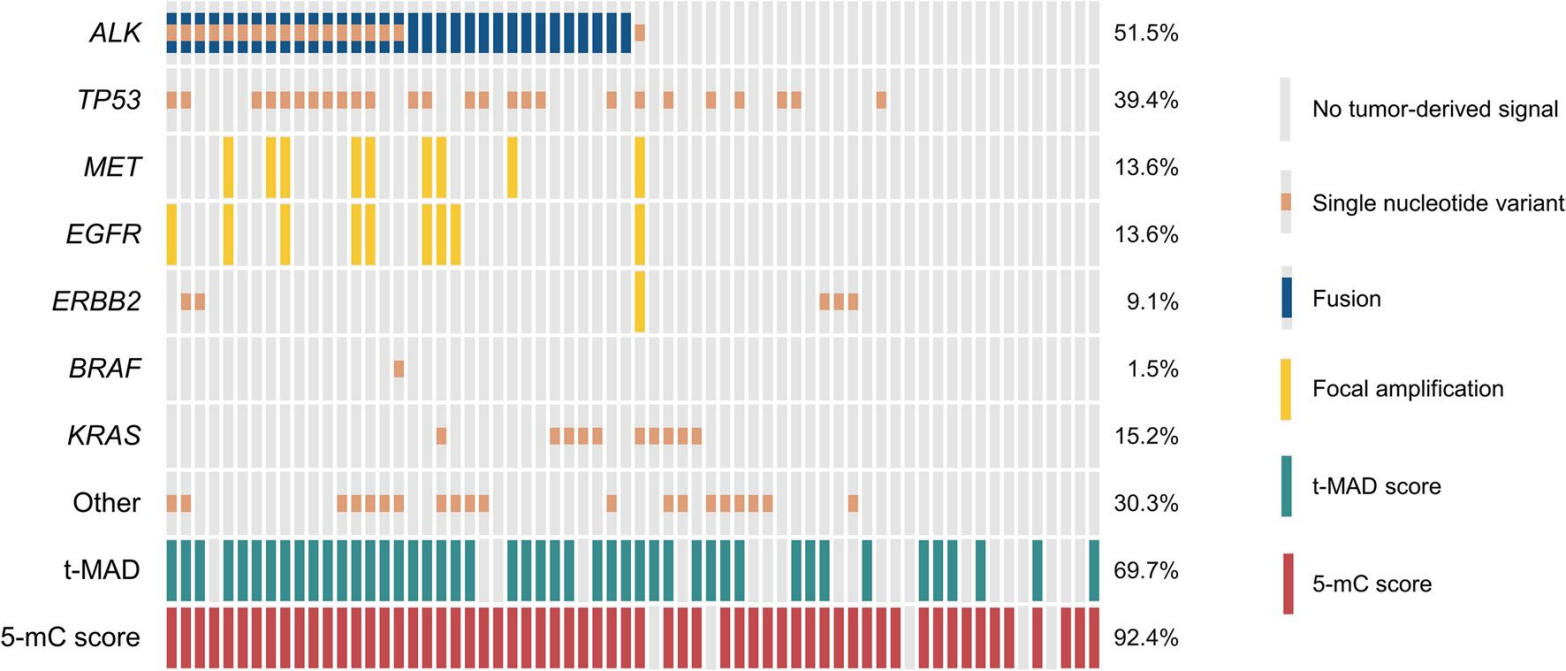
Per-sample OncoPrint of genomic alterations and cancer-derived 5-mC signals. Plasma samples with 5-mC scores exceeding the maximum value in the healthy donor cohort (0.6870) were considered tumor DNA-positive. Positivity for copy number alterations was evaluated similarly (t-MAD score ≥ 0.0081). Mutations, fusions, and focal amplifications with a relative abundance or variant allele frequency ≥ 0.01% were deemed tumor DNA-positive. Percent of tumor DNA-positive samples per biomarker are indicated on the right. *ALK*, anaplastic lymphoma kinase; *BRAF*, B-raf proto-oncogene; *EGFR*, epidermal growth factor receptor; *ERBB2*, Erb-B2 receptor tyrosine kinase 2; *KRAS*, v-Kir-Ras2 Kirsten rat sarcoma viral oncogene homolog; *MET*, MET proto-oncogene; *TP53*, tumor protein 53; t-MAD, trimmed median absolute deviation from copy number neutrality

Cancer-associated alterations of the methylome are prevalent and pervasive across patients [36]. This allows simultaneous profiling of multiple 5-mC biomarkers, potentially overcoming sensitivity limitations posed by the analysis of less abundant genomic alterations. Here, we assessed whether the combined evaluation of the previously identified 245 hyper-DMRs (5-mC score) could identify tumor-derived signals in cfDNA samples without detectable genomic alterations (i.e., t-MAD score, focal amplifications, mutations, or fusions). To define a detection threshold, we calculated 5-mC scores from cfMeDIP-seq data of 13 healthy individuals and used the maximum value (median 0.0224; range 0–0.6870) to determine tumor DNA positivity. The t-MAD score detection threshold was established likewise using sWGS data of 16 healthy individuals (median 0.0051; range 0.0028–0.0081). We identified tumor-derived 5-mC signals in 92.4% (61/66) of cfDNA samples and 90.5% (19/21) of *ALK*-positive patients (Fig. 2 and Additional file 1: Table S4). Hybrid-capture sequencing and sWGS found cancer-associated genomic alterations in 86.4% (57/66) of the samples, constituting 66.7% (14/21) of patients [2, 4]. The most recurrently altered genes were *ALK* (51.5%; 34/66 samples) and *TP53* (39.4%; 26/66 samples). Focal amplifications were detected in 16.7% (11/66) and t-MAD scores exceeding the detection threshold in 69.7% (46/66) of the samples. 5-mC analysis identified tumor-derived methylation changes in 6 samples from 6 patients without reported genomic alterations, whereas tumor DNA in 2 samples (2 patients) was detectable by hybrid-capture sequencing and/or sWGS only. Comparing the 5-mC analysis results to hybrid-capture sequencing or sWGS alone resulted in the identification of 14 and 17 additional samples (14 and 11 patients), respectively, with detectable tumor-derived alterations. Combining all three analysis types, we found tumor DNA in 95.5% (63/66) of all cfDNA samples and in at least one sample in 90.5% (19/21) of patients. This highlighted the added value of a multimodal approach for the detection of cancer signals in cfDNA samples.

### 5-mC score predicts poor overall survival and indicates molecular risk of *ALK*-positive lung cancer

**Fig. 3:**
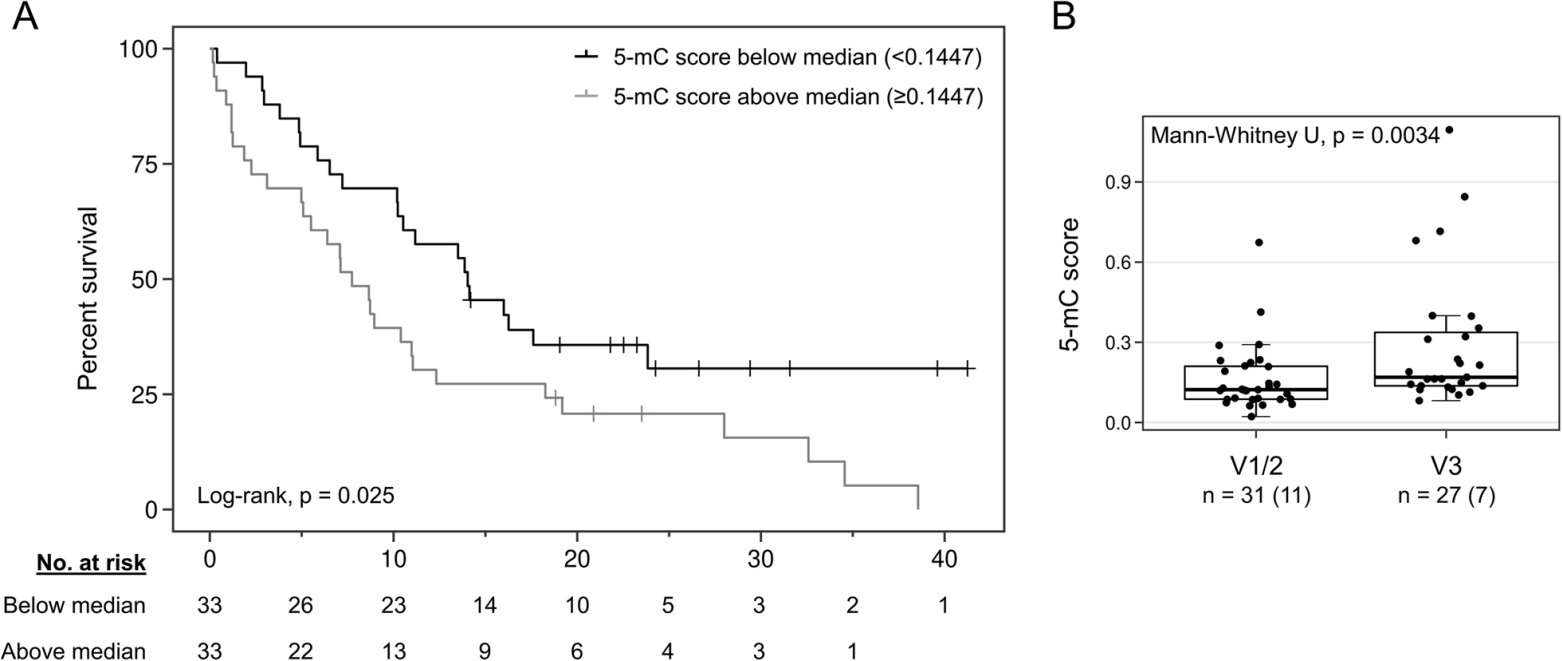
Association of 5-mC scores to overall survival and molecular risk factors. **A** Overall survival of *ALK*-positive NSCLC from the time point of plasma sampling according to the median 5-mC score of all evaluable patient samples (0.1447). **B** 5-mC scores in samples from patients with *EML4-ALK* fusion variant V1/2 and V3 detected in tissue biopsies. Each dot represents one plasma sample, and the number of samples and patients (in brackets) are given per group. Box plot center lines indicate the median, and boxes illustrate the interquartile range with Tukey whiskers. *ALK*, anaplastic lymphoma kinase; *EML4*, echinoderm microtubule-associated protein-like 4

To investigate whether the 5-mC score holds prognostic value, we compared it to the clinical outcomes in our *ALK*-positive patient cohort. Overall survival (OS) from the time of plasma sampling was significantly shorter in cases exceeding the cohort’s median 5-mC score (median 7.7 vs. 14.0 months; *p* = 0.0253; Fig. 3A). *EML4-ALK* fusion abundances and t-MAD scores were also predictive of OS (Additional file 1: Figure S4A/B and previously shown [4]). Recent studies identified the presence of the *EML4-ALK* fusion V3 and/or *TP53* mutations as molecular risk factors associated with shorter progression-free survival and OS in *ALK*-positive patients [37–40]. We found significantly higher 5-mC scores in samples of *EML4*-*ALK* V3 compared to V1/2 patients (*p* = 0.0034; Fig. 3B), while no association between the 5-mC score and *TP53* mutation status was observed (*p* = 0.5543; Additional file 1: Figure S4C). This was in contrast to the elevated *EML4-ALK* fusion levels and higher t-MAD scores detected in both V3 *versus* V1/2 and *TP53*-positive *versus TP53*-negative patients within a previous study [4].

### 5-mC scores reflect disease kinetics under ALK TKI therapy in longitudinal cfDNA samples

**Fig. 4:**
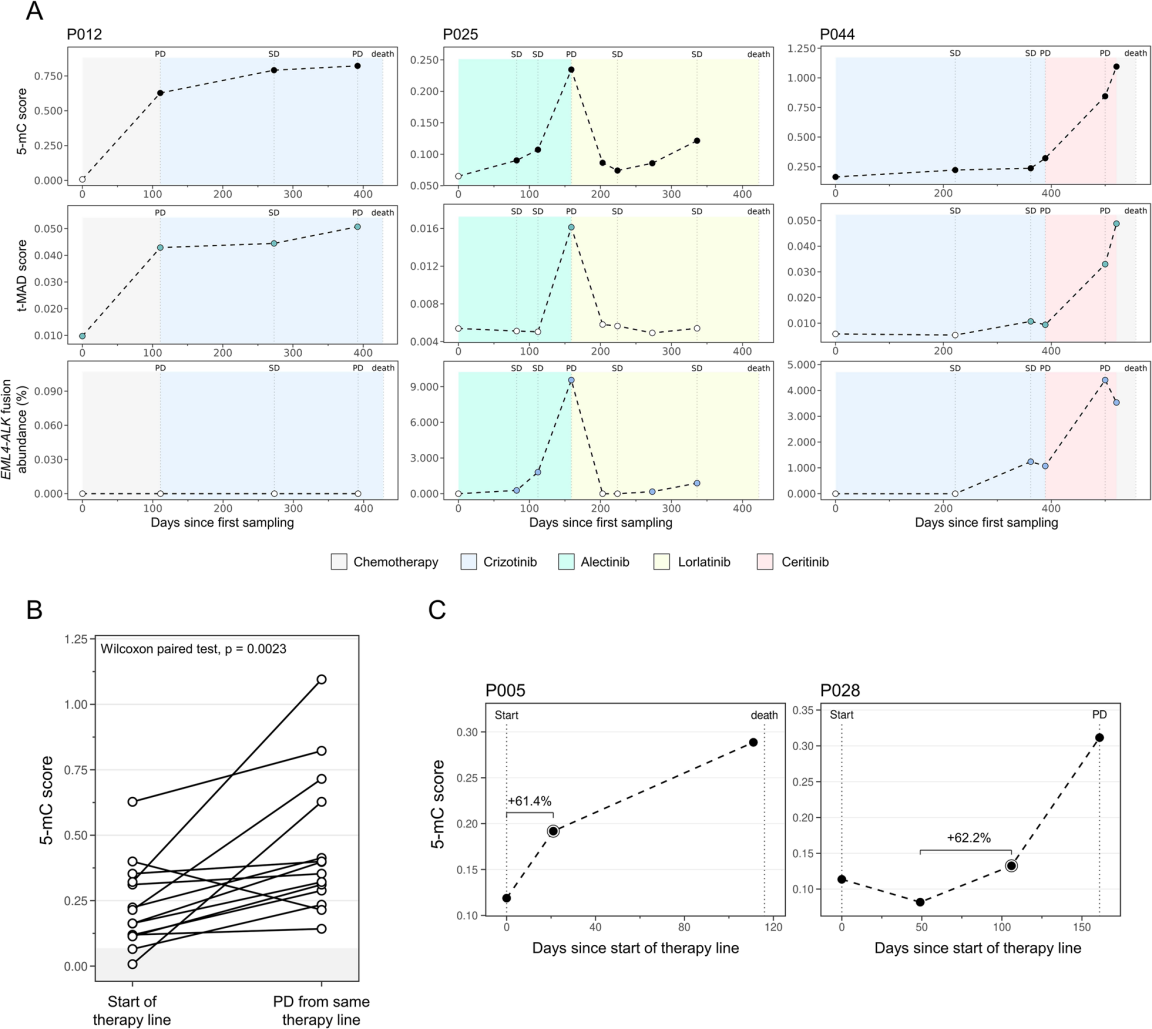
DNA methylation-based therapy monitoring of *ALK*-positive NSCLC patients. **A** Longitudinal cell-free biomarker kinetics of three representative patients. 5-mC scores, t-MAD scores, and EML4-ALK fusion abundances are illustrated from top to bottom. White dots mark data points below the markers respective limit of detection. Therapy regimens are given by the shaded color backgrounds and radiologic disease assessments are highlighted above individual graphs. **B** 5-mC score comparison in cases (*n* = 13) with available plasma samples at therapy line start and at disease progression from the same line. Shaded area illustrates the limit of detection. **C** Representative cases of detectable early molecular progression. Longitudinal profiles show 5-mC score kinetics from therapy line start to disease progression with resultant treatment switch or patient death. Time points of first molecular indication of PD are highlighted, and percent 5-mC score increase from therapy line nadir is illustrated. *ALK*, anaplastic lymphoma kinase; *EML4*, echinoderm microtubule-associated protein-like 4; PD, progressive disease; SD, stable disease

Targeted treatment of *ALK*-positive patients is characterized by high incidences of acquired drug resistance and consequent patient relapse [41, 42]. Regular disease surveillance is therefore instrumental for the early detection of tumor progression and guidance of subsequent therapy decisions. We and others previously demonstrated the feasibility of cfDNA mutation and CNA profiling for the monitoring of *ALK*-positive NSCLC [2, 4, 43–45]. In this study, we assessed whether the 5-mC scores reflected therapy-associated tumor DNA dynamics in the plasma of *ALK*-positive patients. Representative cases of 5-mC-based therapy monitoring are illustrated in Fig. 4A and Additional file 1: Figure S5. 5-mC score kinetics reflected those found in the co-measured cell-free genomic biomarkers and recapitulated radiologic tumor progression. Patients P012, P025, and P044 exemplified cases indicating TKI failure by rising 5-mC scores, while decreasing 5-mC signals after administration of effective therapy regimens were observed in P007, P013, P025, and P028 (Fig. 4A and Additional file 1: Figure S5). Additionally, the cohort included cases without informative *EML4-ALK* fusion abundances (e.g., P012 and P007) or t-MAD scores (P007). 5-mC scores were detectable in both cases and indicated disease progression, highlighting the value of 5-mC profiling. Our cohort comprised 13 instances (in 7 patients) with available plasma at the start of a therapy line and at disease progression from the same line with consequential therapy switch or patient death. Compared to the therapy baseline, 5-mC scores were elevated at the progressive disease (PD) time point in 13/14 cases (Wilcoxon paired test, *p* = 0.0023; Fig. 4B). *EML4-ALK* fusion abundances and t-MAD scores increased at PD in 10/14 and 11/14 cases, respectively (Wilcoxon paired test, *p* = 0.0367 and *p* = 0.0419; Additional file 1: Figure S6A). Interestingly, we observed rising 5-mC scores in samples taken prior to disease progression at radiologically stable disease (SD), potentially marking the development of drug resistance (Fig. 4C and Additional file 1: Figure S5). The plasma sampling scheme of this study allowed detecting early molecular signs of PD at 8 instances (6 patients). Defining a ≥ 25% increase from the therapy line nadir as an indication of molecular progression, we identified 7/8 instances in which 5-mC profiling preceded radiographic determination of TKI failure. The median lead time to radiological progression was 89 days (range 0 to 345 days), allowing significantly earlier relapse identification compared to imaging (Wilcoxon paired test, *p* = 0.0225; Additional file 1: Figure S6B). *EML4-ALK* fusion abundances denoted lead times in 6/8 instances (median 66 days [range 0 to 150 days]; Wilcoxon paired test, *p* = 0.0360) and t-MAD scores were not informative of early molecular progression (Additional file 1: Figure S6B).

### Copy number alteration estimation from cfMeDIP-seq data

The assessment of copy number changes by sWGS of cfDNA demonstrates a cost-effective method for minimally invasive estimation of tumor burden [2, 4, 29, 46]. Here, we evaluated whether cfMeDIP-seq data can be used to infer chromosomal instability, allowing simultaneous genomic and epigenomic tumor assessment from the same dataset. We generated global copy number profiles from cfMeDIP-seq data at 1-Mb bins [47] and compared them to CNAs detected by sWGS of matched plasma. The resulting CNA profiles were highly concordant between both datasets (Additional file 1: Figure S7A). For quantitative CNA detection comparison, we downsampled both datasets to a common read coverage of 5 M paired reads per sample and calculated t-MAD scores for all patient samples. The resulting t-MAD scores were highly correlated (Pearson, *r* = 0.9360; *p* < 2.2e−16; Additional file 1: Figure S7B), showing that cfMeDIP-seq could be used for both 5-mC profiling and genome-wide copy number estimations. In this way, the utility of sequencing data could be increased without additional costs and using fewer resources (i.e., patient material).

## Discussion

In this study, we showed that cancer-derived changes of the methylome can be detected in cfDNA samples of *ALK*-positive NSCLC patients. We implemented a workflow to identify cell-free 5-mC biomarkers, validated their tumor specificity using in-house and external reference datasets, and demonstrated the utility of these markers for prognosis and therapy monitoring.

Tumor-derived DNA can be detected in plasma cfDNA of cancer patients and allows minimally invasive disease assessment [1–7]. Previous reports showed that genome-wide 5-mC profiles can be derived from cfDNA of cancer patients and carry information about the tumor [10, 21–23, 25, 48]. However, a major challenge of liquid biopsies is the detection of minute amounts of tumor DNA molecules within a vast cfDNA background derived from hematopoietic cells. A pivotal element of our 5-mC biomarker identification approach was the initial reduction of 5-mC signals derived from blood cells (Fig. 1A). We found that this step increased the association between *ALK* cfDNA and *ALK* tumor tissue 5-mC signals and therefore reduced the number of genomic regions without tumor-informative DNA methylation. Additionally, the number of DMRs concordantly hypermethylated in cfDNA and tumor tissue increased after the exclusion of the signals derived from hematopoietic cells. This emphasized that the employed background suppression enriches genomic regions containing tumor-derived 5-mC signals and facilitates their detection from cfDNA. Similar observations were made by others showing that the selection of tumor DNA molecules in cfDNA via fragment characteristics (e.g., fragment length) allows the identification of genomic aberrations otherwise missed [29, 49]. Our approach differs from previously reported methods for 5-mC background exclusion [15, 21] in two major points. First, we used public WGBS reference data of individual cell types [18], rather than bulk peripheral blood mononuclear cells, to infer the cfDNA background. Thereby, we were able to account for the relative contribution of each cell type to the cfDNA composition, which has been described to deviate from their abundance in blood [18, 19]. Second, we segmented the genome based on methylation blocks (i.e., adjacent CpG sites with concordant methylation status) [18], instead of continuous 300-bp windows. The assessment of coordinated methylation blocks was reported to reflect fundamental functional 5-mC units and increased the robustness as well as the sensitivity of liquid biopsy assays [9, 50]. Additionally, the evaluation of methylation blocks is well suited to the resolution of cfMeDIP-seq, interrogating 5-mC signals at genomic regions rather than individual CpGs.

Another key advantage of the presented study was the availability of various in-house reference datasets for marker validation (i.e., cfMeDIP-seq data of *ALK* tumor tissue and cell-free genomic alterations determined in matched plasma). The concordance of hyper-DMRs found in both *ALK* cfDNA and cancer tissue confirmed their tumor specificity, which was further validated by the correlation of the 5-mC score to cancer-specific genomic alterations. External datasets (TCGA and GTEx) illustrated the biological plausibility of the identified 5-mC markers and provided insights into transcriptional dysregulation of LUAD-specific genes. Of note, the comparison with TCGA data suggested that many of the cell-free 5-mC marker regions represent methylome alterations found in various molecular LUAD subtypes. Hence, these biomarkers might be applicable to a wider range of patients beyond the *ALK*-positive subtype addressed in this study. Furthermore, some of the 5-mC biomarkers were present in TCGA-LUAD patients with localized disease, indicating their potential applicability for early disease detection. This was corroborated by the identification of methylation-regulated genes (*PCDH10*, *TBX2*, and *CDO1)* recently described as biomarkers in localized lung cancers and premalignant lesions [32–34]. These observations are in line with the early occurrence of 5-mC changes during tumorigenesis and the pervasiveness of 5-mC patterns across tumor types [12, 51–53].

The combined analysis of 5-mC and genomic biomarkers within this study highlighted their complementarity for the detection of tumor-derived DNA in plasma cfDNA samples. We found that 5-mC markers, summarized as the 5-mC score, detected tumor DNA in more samples (*n* = 61) compared to hybrid-capture sequencing (*n* = 49) for mutation analysis or chromosomal instability assessment via the t-MAD score (*n* = 46). A plausible explanation for this finding is the increased number of loci covered (245 tumor-informative 5-mC signals). It has previously been shown that the breadth can supplant the depth of sequencing and increases the sensitivity of liquid biopsy assays [10, 54]. However, 5-mC analysis failed to detect tumor DNA in some samples in which genomic markers were informative and vice versa. In addition, we demonstrated that the 5-mC score is indicative of OS in a per-sample survival analysis. We and others reported similar results for the detectability of tumor DNA by genomic markers (i.e., mutations, *EML4-ALK* fusion or t-MAD scores) [4, 55–57]. In this study, both *EML4-ALK* fusion abundances and t-MAD scores were superior in predicting OS compared to the 5-mC score. This might be explained by their association with *TP53* mutations, a well-described molecular risk factor in *ALK*-positive NSCLC [37, 39], which was not found for the 5-mC score. The high dynamic range of the 5-mC score in *TP53*-negative samples might render 5-mC-based disease assessment more suitable for this patient subgroup instead of the analysis of less abundant genomic markers [4].

Through 5-mC analysis in sequential plasma samples, we highlighted that the 5-mC score reflects tumor dynamics associated with TKI therapy response. We showed that 5-mC signals indicated disease progression and were informative in cases in which the *EML4-ALK* fusion abundance or the t-MAD score remained undetectable. Importantly, we observed several instances of rising 5-mC scores prior to radiologically apparent disease progression which marked early signs of molecular progression. With the high incidence of disease relapse under *ALK* TKI therapy, early identification of disease progression is of particular importance for prolonged therapeutic benefit as we highlighted previously [2].

The main limitations of this study were its retrospective design with heterogeneous sampling time points and administered therapy lines. The varying number of plasma samples per patient might have introduced errors due to the overrepresentation of certain individuals. Corresponding findings should ideally be validated in a larger, prospective study with defined sampling intervals. Moreover, the limited number of samples impeded the definition of robust 5-mC score thresholds, both for the assignment of tumor DNA positivity and for the presence of early molecular progression, and precluded the application of machine learning for 5-mC biomarker identification. In addition, technical restraints of the cfMeDIP-seq method, such as its limited capability to assess DNA hypomethylation, precluded the evaluation of the entire methylome.

## Conclusion

To our knowledge, this is the first study that analyzed 5-mC alterations in cfDNA of *ALK*-rearranged NSCLC and comprehensively monitored their targeted TKI therapy using 5-mC biomarkers. We demonstrated that the employed biomarker identification approach could reliably identify tumor-associated 5-mC signals and might be used as a blueprint for 5-mC marker detection in future studies. We established a quantitative measure for the assessment of cancer-derived 5-mC changes (5-mC score) and demonstrated its suitability for prognostication and longitudinal therapy monitoring.

## Methods

### Patients

All individuals provided written informed consent and the study was approved by the ethics committee at Heidelberg (S-270/2001, S-296/2016, S-435/2019) and Lübeck Universities (AZ 12-238). Patients were screened for *ALK* rearrangements in tissue using at least two of the following approaches: *ALK* immunohistochemistry (D5F3 clone, Roche, Mannheim, Germany), *ALK* fluorescence in situ hybridization (ZytoLight SPEC ALK probe, ZytoVision GmbH, Bremerhaven, Germany), and RNA-based next-generation sequencing (NGS, Thermo Fisher Lung Cancer Fusion Panel, Waltham MA, USA). Plasma samples from 21 *ALK*-positive metastatic NSCLC patients and 13 healthy donors (i.e., subjects without known current disease) were provided by the Lung Biobank Heidelberg, member of the Biomaterial Bank Heidelberg (BMBH), and LungenClinic Grosshansdorf. Serial plasma throughout TKI therapy was available for eleven patients resulting in a total of 79 collected plasma specimens (patient samples, *n* = 66; healthy donor samples, *n* = 13). Additionally, 15 tissue samples from *ALK*-positive metastatic NSCLC patients and 10 distant normal lung tissue specimens (> 5 cm) from NSCLC patients who underwent resection of primary lung cancer at the Thoraxklinik at the University Hospital Heidelberg were provided by the Lung Biobank Heidelberg. For 6 patients, matched plasma and tumor tissue samples were available. The remaining 9 tumor tissue samples were taken from patients not included in the *ALK*-positive cfDNA cohort. All diagnoses were made according to the 2004 WHO classification for lung cancer by at least two experienced pathologists. Tumor histology was classified according to the third edition of the World Health Organization classification system. Clinical data, relevant molecular information (i.e., information about *ALK* fusion variants and *TP53* mutation positivity), and radiographic assessments by chest/abdominal computed tomography and brain magnetic resonance imaging were collected based on patient records with a cutoff on March 3, 2020.

### Blood processing and cfDNA isolation

Peripheral blood was collected in K_2_EDTA tubes and subjected to plasma isolation within one hour of venipuncture employing the previously described centrifugation protocol [4]. Plasma samples were stored at − 80 °C in the Lung Biobank Heidelberg until further processing. cfDNA isolation was performed from 2 mL of plasma using the QIAamp MinElute ccfDNA Kit (Qiagen, Hilden, Germany). The concentration and integrity of cfDNA were assessed by the Qubit dsDNA High Sensitivity Kit (Thermo Fisher Scientific, Waltham MA, USA) and the Bioanalyzer 2100 System with DNA High Sensitivity reagents (Agilent Technologies, Santa Clara CA, USA), respectively.

### Tissue collection and DNA extraction

Tissues were snap-frozen within 30 min after resection and stored at − 80 °C until the time of analysis. For nucleic acid isolation, 10 to 15 tissue cryosections (10 to 15 µm each) were prepared for each patient. The first and the last sections in each series were stained with hematoxylin and eosin (H&E) and tumor samples were reviewed by an experienced lung pathologist to determine the proportions of viable tumor cells, stromal cells, normal lung cell cells, infiltrating lymphocytes and necrotic areas. Only samples with a viable tumor content of ≥ 50% were used for subsequent analyses. Frozen tumor cryosections and matched normal lung tissue pieces were homogenized using the TissueLyser mixer-mill disruptor (2 × 2 min at 25 Hz, Qiagen, Hilden, Germany). Total DNA was isolated with the AllPrep DNA/RNA Universal Kit (Qiagen, Hilden, Germany) following the manufacturer’s instructions. DNA was stored at − 80 °C until further use.

### cfMeDIP-seq library preparation and sequencing

5-mC-enriched sequencing libraries were generated employing a previously published protocol designed for small DNA input quantities (cfMeDIP-seq; [20]). In brief, 2 to 10 ng cfDNA was subjected to end-repair and A-tailing followed by sequencing adapter ligation at 16 °C for 15 h using the KAPA HyperPrep Kit with KAPA Dual-Indexed Adapters (Roche, Mannheim, Germany). Prior to immunoprecipitation, libraries were purified by bead-based double-sided size selection and spiked with methylated and unmethylated control DNA fragments (prepared as described in Song et al*.* [58]) for assessment of 5-mC enrichment efficiency. Additionally, λ phage filler DNA was added to bring the DNA amount to a total of 100 ng. The MagMeDIP qPCR and iPure v2 Kits (Diagenode, Seraing, Belgium) were used for methylation immunoprecipitation and DNA purification, respectively. Enrichment efficiency was assessed by means of qPCR quantification of methylated and unmethylated spike-ins and library amplification was carried out using 12 PCR cycles, followed by another bead clean-up. The final libraries were quantified using the Qubit DNA High Sensitivity Kit and checked for appropriate adapter ligation with the Bioanalyzer 2100 System. Libraries were pooled equimolarly and sequenced in 8-plexes on an Illumina NextSeq550 instrument with high-output reagents (75-bp paired-end reads). Sheared genomic DNA from tissue specimens was processed using the same protocol with two exceptions: (1) 250 ng of DNA input was used per sample and (2) library preparation was performed without the addition of λ phage filler DNA.

Fastq raw reads were adapter trimmed by Cutadapt v3.7 [59] and aligned to the human reference genome GRCh37/hg19 using bowtie2 v2.3.5.1 [60] in paired mode. Aligned reads were indexed, sorted, and filtered by samtools v1.9 [61], retaining only properly paired reads with MAPQ > 10. Duplicate reads were marked with Picard v2.25.1 (MarkDuplicates) and collapsed to allow one read per alignment position. Sequencing data quality was assessed with fastqc v0.11.5 and the MEDIPS R package [62], evaluating coverage saturation (MEDIPS.saturation), CpG enrichment (MEDIPS.CpGenrich), and CpG coverage (MEDIPS.seqCoverage). Per-sample quality metrics are summarized in Additional file 1: Table S5.

### Identification of *ALK*-associated DMRs

Paired fragments were counted by Subread v1.5.3 (featureCounts) [63] at 7,264,350 non-overlapping windows previously described to span CpGs with concordant 5-mC signals (methylation blocks) [18]. Windows covering < 3 CpG sites, mapping to chromosomes X, Y or the mitochondrial genome were excluded. To enrich for windows with cancer-informative 5-mC signals, we inferred regions frequently hypermethylated in plasma cfDNA of healthy individuals and excluded those from further analyses. In brief, whole-genome bisulfite sequencing data of cell types composing healthy donor cfDNA was downloaded (GSE186458 [18] and BLUEPRINT [64]) and processed with the wgbstools suite (https://github.com/nloyfer/wgbs_tools), averaging beta values falling into the same window. Beta values of each cell type were weighted according to their predicted abundance in healthy cfDNA [18] and summed to yield total DNA methylation. Windows with *β* values > 0.15 in the combined dataset were excluded.

To identify *ALK*-associated DMRs, we performed differential methylation analysis between cfDNA from healthy donors (*n* = 13) and *ALK*-positive NSCLC patients (*n* = 21). For patients with longitudinal plasma available, we only considered the sample with the highest t-MAD score, reasoning that these contain elevated quantities of cancer-derived cfDNA. Differential analysis was limited to cancer-informative genomic windows remaining after the filtering steps described before. Additionally, windows with low read counts across all samples were excluded (i.e.,  < 20% of the total number of samples). Trimmed mean of M values (TMM)-normalized counts [65] were subjected to differential methylation analysis using the limma package in R [66]. Following variance smoothing, a linear model using weighted least squares was fit for each genomic region. *P* values between cancer and control conditions were calculated by empirical Bayes smoothing. Significantly hyper- or hypomethylated regions were called at adjusted *p* values (Benjamini-Hochberg) < 0.1 and |log_2_FC|> 1. *ALK* tissue DMRs (i.e., *ALK* tissues *vs.* normal lung tissues) were identified likewise, omitting the exclusion of genomic regions hypermethylated in healthy plasma cfDNA.

### 5-mC score calculation

Aligned bam files of 13 healthy control samples were downsampled to a common read coverage and merged, yielding a combined read depth of 28 million paired-reads (median read depth across all cfDNA cfMeDIP-seq datasets). The resulting normal reference file was used as a baseline to quantitatively assess the extent of cancer-derived 5-mC changes in our patient plasma samples. The median absolute RPKM (reads per kilobase per million mapped reads) deviation from this baseline at relevant hyper-DMRs was calculated per sample and defined as ‘5-mC score.’

### Processing of publicly available DNA methylation and gene expression data of tissue samples

Illumina 450 k methylation array and RNA sequencing data of primary tumor tissues from lung adenocarcinoma patients—alongside various adjacent normal tissues (breast, bladder, colon, endometrium, head and neck, kidney, liver, lung, prostate, and thyroid gland)—was obtained from TCGA [30, 31]. Additional gene expression data of normal lung tissues (*n* = 288) were retrieved from the GTEx [67]. All datasets, alongside clinical and molecular annotations, were downloaded from the Xena platform [68]. DNA methylation array data were adjusted to the genomic regions used for DMR calling from cfMeDIP-seq datasets by averaging *β* values of CpGs falling into the same region. Differential methylation analysis between LUAD (*n* = 455) and normal lung tissue samples (*n* = 75; taken from LUAD and LUSC cohorts) was performed using limma [66], only considering genomic regions with healthy cfDNA 5-mC signals *β* ≤ 0.15. Regions with |∆*β* ≥ 0.25|and adjusted *p* value < 0.01 were deemed significantly hyper- or hypomethylated. Further differential methylation analyses, stratifying patient samples by pathologic stage and molecular driver event, are summarized in Additional file 1: Table S3. *ALK*-positive patients within the TCGA-LUAD cohort were determined using the TumorFusions data portal [69]. To identify genomic regions with gene regulatory 5-mC signals, we correlated matched DNA methylation and gene expression data of adjacent normal tissues (*n* = 15 per tissue type). 5-mC signals demonstrating a significant negative correlation (Spearman, *p* < 0.05) to the expression level of its associated gene were considered to be involved in transcriptional regulation. Genomic feature annotation was performed using the annotatr R package [70].

### Genomic cfDNA biomarkers

Information on cancer-specific genomic alterations of the patient plasma samples profiled in this study was obtained from previously published work (for detailed descriptions see [2, 4]). Somatic mutations and *EML4-ALK* fusion abundances were determined by hybrid-capture sequencing using the AVENIO ctDNA Library Preparation Kit followed by sequencing with the Targeted or Surveillance Panel (Roche, Mannheim, Germany). Mutations with variant allele frequencies (VAFs)≥ 30% were considered germline mutations and consequentially excluded from further analyses. VAFs < 0.01% were deemed undetectable. Genome-wide copy number profiles and t-MAD scores were estimated from sWGS data using the ichorCNA algorithm [47] and t-MAD score calculation documentation (https://github.com/sdchandra/tMAD) [29], respectively. CNA calling was carried out at 1-Mb bin sizes using sWGS data of 16 healthy control samples as copy number neutral references. The sequencing data was downsampled to 5 M paired reads prior to t-MAD score calculation. The maximal t-MAD score across all healthy control samples (0.0081) was set as the detection threshold. Chromosomal instabilities were similarly assessed from cfMeDIP-seq data with 5-mC-enriched sequencing data of healthy controls (*n* = 13) as copy number neutral reference for normalization.

### Statistical analyses and data visualization

A comparison between independent and paired data was made using the Mann–Whitney *U* test and Wilcoxon’s paired test, respectively (as labeled in graphs). Spearman’s correlation was used to test the association between *ALK* cfDNA and *ALK* tissue 5-mC signals as well as 5-mC scores and genomic biomarker abundances. T-MAD scores inferred from sWGS and cfMeDIP-seq were compared by Pearson’s correlation. Permutation testing to estimate the significance of the overlap between tissue and cfDNA DMRs was performed with the regioneR R package [71], comparing the observed overlap to a null distribution of 10,000 random samplings. The permutation test p value represents the number of random samplings with overlaps greater or equal to the observed overlap divided by the number of random permutations. Survival data were analyzed according to Kaplan–Meier using the log-rank test for OS comparison. Statistical analyses were performed in R (version 3.6.2) [72] and relevant graphs were generated using the ggplot2 R package [73].

## Supplementary Information

**Additional file 1:** Supplementary results.

## Data Availability

All sequencing data generated in this study are deposited in the European Genome-Phenome Archive (EGA) under accession number EGAS00001006573. TCGA Illumina 450 k methylation array and RNA sequencing data are publicly available through the Genomics Data Commons (https://portal.gdc.cancer.gov) and the GTEx portal (https://gtexportal.org). For this study, data were downloaded as uniformly preprocessed datasets from the Xena platform (https://xenabrowser.net). WGBS data of hematopoietic cell types, vascular endothelial cells, and hepatocytes were downloaded from the Gene Expression Omnibus (GSE186458). The code used to process cfMeDIP-seq data, identify 5-mC biomarkers, and calculate 5-mC scores can be obtained from the authors upon reasonable request.
