# Supplementary material for: Longitudinal monitoring of cell-free DNA methylation in ALK-positive non-small cell lung cancer patients
Source: Clin Epigenetics. 2022 Dec 2;14:163. doi: 10.1186/s13148-022-01387-4 (PMC9719130; doi:10.1186/s13148-022-01387-4)
Supplement: Supplementary file 1 — Additional file 1: Supplementary results. [file 13148_2022_1387_MOESM1_ESM.docx]

# Supplementary tables

**Table S1: Per sample demographic and molecular cohort information.**

| **Sample ID** | **Patient ID** | **Gender** | **Age** | **Pathologic stage** | **EML4-ALK fusion variant** | **TP53 mutation status** | **EML4-ALK fusion abundance (%)** | **t-MAD score** | **Sampling date** | **Last follow-up date** | **Deceased at last follow-up (Y/N)** | **Overall survival from sampling time point (days)** | **Included for 5-mC marker identification (Y/N)** |
| --- | --- | --- | --- | --- | --- | --- | --- | --- | --- | --- | --- | --- | --- |
| P001_01 | P001 | male | 54 | Stage IV | V3 | positive | ND | 0.0069 | 12/5/2017 | 7/9/2019 | N | 581 | Y |
| P002_01 | P002 | female | 51 | Stage IV | V1 | negative | ND | 0.0070 | 2/6/2017 | 9/27/2019 | N | 963 | Y |
| P003_01 | P003 | male | 46 | Stage IV | E9/10 | positive | 0.19 | 0.0136 | 10/12/2017 | 3/9/2018 | Y | 148 | Y |
| P003_05 | P003 | male | 46 | Stage IV | E9/10 | positive | ND | 0.0091 | 8/1/2016 | 3/9/2018 | Y | 585 | N |
| P003_09 | P003 | male | 46 | Stage IV | E9/10 | positive | ND | 0.0094 | 1/8/2018 | 3/9/2018 | Y | 60 | N |
| P005_01 | P005 | male | 56 | Stage IV | V1 | positive | 4.84 | 0.0287 | 12/8/2015 | 12/13/2015 | Y | 5 | Y |
| P005_03 | P005 | male | 56 | Stage IV | V1 | positive | 0.14 | 0.0087 | 8/19/2015 | 12/13/2015 | Y | 116 | N |
| P005_04 | P005 | male | 56 | Stage IV | V1 | positive | 0.24 | 0.0051 | 9/9/2015 | 12/13/2015 | Y | 95 | N |
| P007_01 | P007 | female | 56 | Stage IV | V2 | negative | ND | 0.0074 | 8/12/2016 | 1/22/2020 | N | 1258 | N |
| P007_02 | P007 | female | 56 | Stage IV | V2 | negative | ND | 0.0554 | 8/8/2017 | 1/22/2020 | N | 897 | Y |
| P007_05 | P007 | female | 56 | Stage IV | V2 | negative | ND | 0.0082 | 11/15/2018 | 1/22/2020 | N | 433 | N |
| P010_01 | P010 | female | 68 | Stage IV | V3 | negative | ND | 0.0162 | 11/7/2017 | 8/6/2019 | N | 637 | Y |
| P010_02 | P010 | female | 68 | Stage IV | V3 | negative | ND | 0.0077 | 1/9/2018 | 8/6/2019 | N | 574 | N |
| P012_02 | P012 | female | 67 | Stage IV | K9A20 | negative | ND | 0.0506 | 2/2/2016 | 7/6/2016 | Y | 155 | Y |
| P012_03 | P012 | female | 67 | Stage IV | K9A20 | negative | ND | 0.0488 | 5/31/2016 | 7/6/2016 | Y | 36 | N |
| P012_04 | P012 | female | 67 | Stage IV | K9A20 | negative | ND | 0.0098 | 5/5/2015 | 7/6/2016 | Y | 428 | N |
| P012_05 | P012 | female | 67 | Stage IV | K9A20 | negative | ND | 0.0394 | 8/24/2015 | 7/6/2016 | Y | 317 | N |
| P013_01 | P013 | female | 53 | Stage IV | V1 | negative | 0.65 | 0.0083 | 5/23/2017 | 9/24/2019 | Y | 854 | N |
| P013_02 | P013 | female | 53 | Stage IV | V1 | negative | 0.04 | 0.0097 | 9/27/2017 | 9/24/2019 | Y | 727 | N |
| P013_03 | P013 | female | 53 | Stage IV | V1 | negative | 2.05 | 0.0132 | 1/3/2017 | 9/24/2019 | Y | 994 | Y |
| P013_05 | P013 | female | 53 | Stage IV | V1 | negative | ND | 0.0046 | 5/16/2018 | 9/24/2019 | Y | 496 | N |
| P013_06 | P013 | female | 53 | Stage IV | V1 | negative | 0.73 | 0.0087 | 7/5/2016 | 9/24/2019 | Y | 1176 | N |
| P013_07 | P013 | female | 53 | Stage IV | V1 | negative | 0.29 | 0.0069 | 11/4/2016 | 9/24/2019 | Y | 1054 | N |
| P013_10 | P013 | female | 53 | Stage IV | V1 | negative | ND | 0.0067 | 8/8/2018 | 9/24/2019 | Y | 412 | N |
| P013_11 | P013 | female | 53 | Stage IV | V1 | negative | ND | 0.0055 | 11/7/2018 | 9/24/2019 | Y | 321 | N |
| P013_12 | P013 | female | 53 | Stage IV | V1 | negative | 0.26 | 0.0072 | 2/20/2019 | 9/24/2019 | Y | 216 | N |
| P014_01 | P014 | male | 53 | Stage IV | NA | NA | ND | 0.0061 | 10/27/2017 | 8/23/2019 | N | 665 | Y |
| P015_01 | P015 | female | 48 | Stage IV | V1 | negative | ND | 0.0064 | 12/4/2017 | 10/22/2019 | N | 687 | Y |
| P019_01 | P019 | female | 59 | Stage IV | V3 | positive | 1.28 | 0.0171 | 6/13/2016 | 7/21/2016 | Y | 38 | Y |
| P019_02 | P019 | female | 59 | Stage IV | V3 | positive | 0.47 | 0.0085 | 5/13/2016 | 7/21/2016 | Y | 69 | N |
| P020_01 | P020 | male | 64 | Stage IV | V1 | negative | 5.94 | 0.0207 | 10/20/2016 | 10/27/2016 | Y | 7 | Y |
| P020_02 | P020 | male | 64 | Stage IV | V1 | negative | ND | 0.0069 | 1/28/2016 | 10/27/2016 | Y | 273 | N |
| P021_02 | P021 | female | 80 | Stage IV | V2 | positive | ND | 0.0086 | 4/7/2016 | 7/29/2019 | N | 1208 | Y |
| P025_01 | P025 | male | 52 | Stage IV | V1 | negative | ND | 0.0128 | 9/27/2017 | 11/24/2018 | Y | 423 | N |
| P025_02 | P025 | male | 52 | Stage IV | V1 | negative | 0.28 | 0.0091 | 12/18/2017 | 11/24/2018 | Y | 341 | N |
| P025_03 | P025 | male | 52 | Stage IV | V1 | negative | 1.81 | 0.0085 | 1/17/2018 | 11/24/2018 | Y | 311 | N |
| P025_04 | P025 | male | 52 | Stage IV | V1 | negative | 9.56 | 0.0177 | 3/5/2018 | 11/24/2018 | Y | 264 | Y |
| P025_05 | P025 | male | 52 | Stage IV | V1 | negative | ND | 0.0071 | 4/18/2018 | 11/24/2018 | Y | 220 | N |
| P025_06 | P025 | male | 52 | Stage IV | V1 | negative | ND | 0.0095 | 5/9/2018 | 11/24/2018 | Y | 199 | N |
| P025_07 | P025 | male | 52 | Stage IV | V1 | negative | 0.17 | 0.0062 | 6/27/2018 | 11/24/2018 | Y | 150 | N |
| P025_08 | P025 | male | 52 | Stage IV | V1 | negative | 0.89 | 0.0122 | 8/29/2018 | 11/24/2018 | Y | 87 | N |
| P027_02 | P027 | male | 51 | Stage IV | V1 | negative | ND | 0.0090 | 9/27/2017 | 10/7/2019 | N | 740 | Y |
| P028_01 | P028 | female | 57 | Stage IV | V3 | positive | 3.98 | 0.0426 | 1/4/2018 | 1/15/2019 | Y | 376 | N |
| P028_02 | P028 | female | 57 | Stage IV | V3 | positive | 5.54 | 0.0286 | 2/12/2018 | 1/15/2019 | Y | 337 | N |
| P028_03 | P028 | female | 57 | Stage IV | V3 | positive | ND | 0.0087 | 7/27/2017 | 1/15/2019 | Y | 537 | N |
| P028_04 | P028 | female | 57 | Stage IV | V3 | positive | ND | 0.0091 | 9/14/2017 | 1/15/2019 | Y | 488 | N |
| P028_05 | P028 | female | 57 | Stage IV | V3 | positive | 1.00 | 0.0091 | 11/10/2017 | 1/15/2019 | Y | 431 | N |
| P028_06 | P028 | female | 57 | Stage IV | V3 | positive | 1.62 | 0.0140 | 3/9/2018 | 1/15/2019 | Y | 312 | N |
| P028_07 | P028 | female | 57 | Stage IV | V3 | positive | 9.66 | 0.0439 | 4/24/2018 | 1/15/2019 | Y | 266 | N |
| P028_08 | P028 | female | 57 | Stage IV | V3 | positive | 2.36 | 0.0175 | 5/24/2018 | 1/15/2019 | Y | 236 | N |
| P028_09 | P028 | female | 57 | Stage IV | V3 | positive | 1.49 | 0.0220 | 6/12/2018 | 1/15/2019 | Y | 217 | N |
| P028_10 | P028 | female | 57 | Stage IV | V3 | positive | 1.23 | 0.0163 | 7/20/2018 | 1/15/2019 | Y | 179 | N |
| P028_11 | P028 | female | 57 | Stage IV | V3 | positive | 1.47 | 0.0233 | 8/16/2018 | 1/15/2019 | Y | 152 | N |
| P028_12 | P028 | female | 57 | Stage IV | V3 | positive | 0.61 | 0.0097 | 10/17/2018 | 1/15/2019 | Y | 90 | N |
| P028_13 | P028 | female | 57 | Stage IV | V3 | positive | 9.95 | 0.1091 | 12/19/2018 | 1/15/2019 | Y | 27 | N |
| P028_14 | P028 | female | 57 | Stage IV | V3 | positive | 12.99 | 0.1282 | 1/4/2019 | 1/15/2019 | Y | 11 | Y |
| P031_01 | P031 | female | 44 | Stage IV | V1 | negative | ND | 0.0069 | 10/10/2017 | 9/19/2019 | N | 709 | Y |
| P032_01 | P032 | male | 65 | Stage IV | V1 | negative | ND | 0.0049 | 10/23/2017 | 10/10/2019 | N | 717 | Y |
| P037_01 | P037 | male | 42 | Stage IV | V3 | negative | ND | 0.0054 | 11/13/2017 | 2/3/2020 | N | 812 | Y |
| P044_02 | P044 | male | 60 | Stage IV | V3 | negative | ND | 0.0080 | 11/28/2014 | 6/7/2016 | Y | 557 | N |
| P044_03 | P044 | male | 60 | Stage IV | V3 | negative | ND | 0.0077 | 7/8/2015 | 6/7/2016 | Y | 335 | N |
| P044_04 | P044 | male | 60 | Stage IV | V3 | negative | 1.24 | 0.0101 | 11/25/2015 | 6/7/2016 | Y | 195 | N |
| P044_05 | P044 | male | 60 | Stage IV | V3 | negative | 1.07 | 0.0103 | 12/22/2015 | 6/7/2016 | Y | 168 | N |
| P044_06 | P044 | male | 60 | Stage IV | V3 | negative | 4.40 | 0.0375 | 4/11/2016 | 6/7/2016 | Y | 57 | N |
| P044_07 | P044 | male | 60 | Stage IV | V3 | negative | 3.53 | 0.0491 | 5/2/2016 | 6/7/2016 | Y | 36 | Y |
| P046_01 | P046 | male | 58 | Stage IV | V3 | negative | ND | 0.0084 | 2/16/2015 | 2/28/2015 | Y | 12 | Y |

*ALK*, anaplastic lymphoma kinase; *EML4*, echinoderm microtubule-associated protein-like 4; NA, information not available; ND, not detected; *TP53*, tumor protein 53; t-MAD, trimmed median absolute deviation from copy number neutrality.

###

**Table S2: Differential methylation analysis results comparing TCGA-LUAD primary tumor *versus* normal adjacent lung tissue.**

| **Tumor sample stratification** | **#Primary tumor tissue samples** | **#Adjacent normal lung tissues** | **#hyper-methylated regions** | **Overlap w/ *ALK*-specific hyper-DMRs** | **Significance of overlap** |
| --- | --- | --- | --- | --- | --- |
| No stratification | 455 | 75 | 2052 | 78/189 | p < 0.0001 |
| Stage I | 250 | 75 | 1905 | 71/189 | p < 0.0001 |
| Stage II | 112 | 75 | 2284 | 82/189 | p < 0.0001 |
| Stage III | 73 | 75 | 2437 | 96/189 | p < 0.0001 |
| Stage IV | 20 | 75 | 1963 | 79/189 | p < 0.0001 |
| *EGFR* | 33 | 75 | 2570 | 92/189 | p < 0.0001 |
| *KRAS* | 68 | 75 | 2938 | 109/189 | p < 0.0001 |
| *ALK* | 5 | 75 | 1677 | 51/189 | p < 0.0001 |

Overlap of hyper-DMRs identified in *ALK*-positive cfDNA (*vs.* healthy donor cfDNA) and primary tumor tissue of TCGA-LUAD samples (*vs.* adjacent normal lung tissue). TCGA-LUAD samples were stratified according to pathologic stage and molecular driver event. Significance of overlap was assessed by permutation testing.

*ALK*, anaplastic lymphoma kinase; DMR, differentially methylated region; *EGFR*, epidermal growth factor receptor; *KRAS*, v-Kir-Ras2 Kirsten rat sarcoma viral oncogene homolog; LUAD, lung adenocarcinoma; TCGA, The Cancer Genome Atlas.

###

**Table S3: Genomic regions with cell-free 5-mC signals indicative of tumor tissue gene expression.**

| **Genomic region** | **Chromo-some** | **Start position** | **End position** | **Associated gene** | **Genomic feature** | **CpG association** | **Functional methylation (Spearman, rho)** | **Differential methylation** | | **Differential gene expression** | |
| --- | --- | --- | --- | --- | --- | --- | --- | --- | --- | --- | --- |
|  |  |  |  |  |  |  |  | **log2(fold-change)** | **adjusted**  **p-value** | **log2(fold-change)** | **adjusted**  **p-value** |
| bin_113178 | chr1 | 29804977 | 29805239 | *PTPRU* | intergenic | island | -0.276 | 1.20 | 2.6E-02 | 0.03 | 3E-02 |
| bin_165486 | chr1 | 47697534 | 47698127 | *TAL1* | promoter | island | -0.217 | 1.15 | 3.1E-02 | -4.63 | 3E-119 |
| bin_165501 | chr1 | 47701123 | 47701242 | *TAL1* | promoter | shelf | -0.324 | 1.13 | 5.1E-02 | -4.63 | 3E-119 |
| bin_1670591 | chr4 | 44449271 | 44450121 | *KCTD8* | intron | island | -0.534 | 1.44 | 1.6E-02 | -0.86 | 3E-06 |
| bin_1839272 | chr4 | 134071952 | 134072602 | *PCDH10* | promoter | island | -0.241 | 1.90 | 5.3E-05 | -2.55 | 3E-66 |
| bin_1839275 | chr4 | 134073584 | 134073906 | *PCDH10* | promoter | island | -0.307 | 1.19 | 2.9E-02 | -2.55 | 3E-66 |
| bin_1921168 | chr4 | 174443713 | 174444041 | *HAND2* | intergenic | island | -0.334 | 1.51 | 2.7E-03 | -4.70 | 6E-78 |
| bin_2216463 | chr5 | 115151388 | 115152432 | *CDO1* | promoter | island | -0.357 | 1.87 | 2.5E-05 | -3.70 | 2E-111 |
| bin_2260979 | chr5 | 136834090 | 136834847 | *SPOCK1* | promoter | island | -0.490 | 1.63 | 2.4E-03 | -0.41 | 3E-04 |
| bin_2375860 | chr5 | 178367770 | 178368206 | *ZNF454* | promoter | island | -0.588 | 1.30 | 4.2E-02 | -1.15 | 8E-22 |
| bin_2457841 | chr6 | 28175416 | 28175922 | *TOB2P1* | promoter | island | -0.269 | 1.05 | 9.2E-02 | 2.50 | 3E-50 |
| bin_2616828 | chr6 | 100912089 | 100912448 | *SIM1* | promoter | island | -0.252 | 1.41 | 1.1E-02 | 1.77 | 1E-19 |
| bin_2778988 | chr7 | 1279649 | 1280147 | *UNCX* | intergenic | island | -0.214 | 1.23 | 7.6E-02 | -0.22 | 7E-06 |
| bin_2845050 | chr7 | 27204764 | 27205249 | *HOXA9* | promoter | island | -0.454 | 1.19 | 1.9E-02 | -1.37 | 4E-38 |
| bin_2845052 | chr7 | 27205266 | 27205723 | *HOXA9* | promoter | island | -0.621 | 1.68 | 3.9E-04 | -1.37 | 4E-38 |
| bin_2845188 | chr7 | 27237766 | 27238129 | *HOXA13* | promoter | shore | -0.372 | 1.49 | 1.1E-02 | 1.98 | 9E-15 |
| bin_3385723 | chr8 | 85095812 | 85096215 | *RALYL* | promoter | shore | -0.249 | 1.20 | 7.5E-02 | -1.47 | 2E-15 |
| bin_4254403 | chr11 | 30037875 | 30038135 | *KCNA4* | promoter | island | -0.264 | 1.16 | 8.7E-02 | -4.03 | 7E-82 |
| bin_4259716 | chr11 | 32455620 | 32455858 | *WT1* | promoter | island | -0.216 | 1.29 | 2.9E-02 | 0.29 | 4E-01 |
| bin_4651877 | chr12 | 54379415 | 54379790 | *HOXC10* | exon | island | -0.401 | 1.24 | 1.4E-02 | 3.58 | 1E-23 |
| bin_4671041 | chr12 | 62584898 | 62585442 | *FAM19A2* | promoter | island | -0.264 | 1.08 | 4.2E-02 | -1.03 | 4E-39 |
| bin_4677031 | chr12 | 65672091 | 65672211 | *MSRB3* | promoter | shore | -0.236 | 1.23 | 6.2E-02 | -2.47 | 3E-110 |
| bin_4947929 | chr13 | 58204002 | 58204322 | *PCDH17* | promoter | island | -0.273 | 1.19 | 4.4E-02 | -1.97 | 2E-76 |
| bin_5658370 | chr16 | 51187536 | 51187808 | *SALL1* | promoter | island | -0.201 | 1.27 | 6.6E-02 | 1.38 | 3E-06 |
| bin_5658380 | chr16 | 51188664 | 51188718 | *SALL1* | promoter | island | -0.248 | 1.23 | 7.8E-02 | 1.38 | 3E-06 |
| bin_5955337 | chr17 | 59479183 | 59479463 | *TBX2* | intron | island | -0.329 | 1.46 | 1.1E-02 | -4.04 | 4E-117 |
| bin_6274422 | chr19 | 11785079 | 11785230 | *ZNF833P* | intron | island | -0.303 | 1.29 | 2.5E-02 | -1.11 | 5E-44 |
| bin_6352671 | chr19 | 36736089 | 36736708 | *ZNF565* | intergenic | island | -0.298 | 1.64 | 5.0E-03 | -0.11 | 5E-05 |
| bin_6413263 | chr19 | 52956657 | 52957158 | *ZNF578* | promoter | island | -0.574 | 1.43 | 2.1E-02 | -1.39 | 2E-44 |
| bin_6413547 | chr19 | 53038825 | 53039605 | *ZNF808* | intron | island | -0.312 | 1.19 | 5.3E-02 | -0.44 | 1E-24 |
| bin_6415343 | chr19 | 53757551 | 53758310 | *ZNF677* | promoter | island | -0.602 | 1.21 | 8.8E-02 | -1.45 | 6E-48 |
| bin_810830 | chr2 | 105478267 | 105478701 | *POU3F3* | intergenic | island | -0.330 | 1.34 | 1.1E-02 | -1.35 | 2E-17 |
| bin_830532 | chr2 | 114034478 | 114034600 | *PAX8* | intron | island | -0.493 | 1.04 | 8.8E-02 | -1.15 | 2E-21 |
| bin_925981 | chr2 | 155555233 | 155555584 | *KCNJ3* | promoter | island | -0.283 | 1.23 | 2.4E-02 | -2.12 | 2E-28 |
| bin_967572 | chr2 | 176993545 | 176994048 | *HOXD8* | promoter | island | -0.308 | 1.18 | 2.4E-02 | 0.10 | 9E-01 |

List of genomic regions alongside their neighboring genes. Genomic feature and CpG annotations were generated using the annotatr R package. ‘Promoter’ refers to regions ≤ 5kb upstream of the transcription start site and 5’-untranslated regions. Spearman correlation coefficients between matched RNA-seq and Illumina 450k methylation array data of various TCGA normal tissues (n = 150 samples) indicate 5-mC-dependent transcriptional repression. Differential methylation refers to the comparison between *ALK*-positive and healthy cfDNA (cfMeDIP-seq) generated within this study. Differential gene expression compares TCGA-LUAD (n = 507) to GTEx normal lung expression data (n = 288).

ALK, anaplastic lymphoma kinase; cfDNA, cell-free DNA; CpG, cytosine-guanine dinucleotide; GTEx, The Genotype-Tissue Expression project; LUAD, lung adenocarcinoma; TCGA, The Cancer Genome Atlas.

###

**Table S4: Tumor DNA detectability comparison between hybrid-capture sequencing, sWGS and cfMeDIP-seq.**

|  |  | **Hybrid-capture sequencing**  **(AVENIO panel)** | **sWGS**  **(t-MAD score)** | **cfMeDIP-seq (5-mC score)** | **Combined** |
| --- | --- | --- | --- | --- | --- |
| Positive samples | n | 49 | 46 | 61 | 63 |
| n = 66 | % | 74.2 | 69.7 | 92.4 | 95.5 |
| Positive patients | n | 10 | 14 | 19 | 19 |
| n = 21 | % | 47.6 | 66.7 | 90.5 | 90.5 |

cfMeDIP-seq, cell-free methylation DNA immunoprecipitation sequencing; sWGS, shallow whole genome sequencing; t-MAD, trimmed median absolute deviation from copy number neutrality.

###

**Table S5: Sequencing data quality control summary.**

| **Sample ID** | **Specimen** | **DNA input (ng)** | **#Raw paired reads (x1e06)** | **#Filtered paired reads (x1e06)** | **Filter-passing rate (%)** | **Coverage saturation (estimated Pearson, r)** | **CpG enrichment score** | **CpGs covered (%)** | **CpGs covered >5 times (%)** | **Reads without CpG (%)** |
| --- | --- | --- | --- | --- | --- | --- | --- | --- | --- | --- |
| P001_01 | Plasma | 4.3 | 51.22 | 18.07 | 0.35 | 0.99 | 3.18 | 0.60 | 0.25 | 0.02 |
| P002_01 | Plasma | 5.3 | 47.60 | 20.11 | 0.42 | 0.98 | 3.41 | 0.58 | 0.23 | 0.02 |
| P003_01 | Plasma | 3.9 | 53.02 | 18.96 | 0.36 | 0.99 | 2.94 | 0.63 | 0.27 | 0.01 |
| P003_05 | Plasma | 10.0 | 63.47 | 35.52 | 0.56 | 0.98 | 3.06 | 0.71 | 0.32 | 0.05 |
| P003_09 | Plasma | 3.3 | 59.91 | 21.99 | 0.37 | 0.98 | 2.84 | 0.68 | 0.28 | 0.03 |
| P005_01 | Plasma | 10.0 | 31.01 | 21.85 | 0.70 | 0.98 | 3.03 | 0.72 | 0.35 | 0.05 |
| P005_03 | Plasma | 10.0 | 53.55 | 31.84 | 0.59 | 0.98 | 3.11 | 0.67 | 0.29 | 0.05 |
| P005_04 | Plasma | 10.0 | 53.70 | 29.95 | 0.56 | 0.97 | 3.08 | 0.68 | 0.27 | 0.06 |
| P007_01 | Plasma | 10.0 | 59.96 | 38.10 | 0.64 | 0.98 | 2.94 | 0.73 | 0.34 | 0.05 |
| P007_02 | Plasma | 10.0 | 53.44 | 33.16 | 0.62 | 0.98 | 3.04 | 0.69 | 0.32 | 0.04 |
| P007_05 | Plasma | 4.4 | 55.94 | 30.98 | 0.55 | 0.98 | 3.07 | 0.70 | 0.31 | 0.03 |
| P010_01 | Plasma | 6.7 | 65.90 | 34.49 | 0.52 | 0.99 | 3.00 | 0.72 | 0.36 | 0.02 |
| P012_02 | Plasma | 10.0 | 53.85 | 34.01 | 0.63 | 0.98 | 2.90 | 0.74 | 0.35 | 0.03 |
| P012_03 | Plasma | 10.0 | 56.67 | 34.52 | 0.61 | 0.98 | 2.87 | 0.72 | 0.34 | 0.05 |
| P012_04 | Plasma | 2.5 | 45.63 | 16.02 | 0.35 | 0.98 | 3.29 | 0.57 | 0.21 | 0.02 |
| P012_05 | Plasma | 7.6 | 59.72 | 30.54 | 0.51 | 0.98 | 3.06 | 0.70 | 0.34 | 0.02 |
| P013_01 | Plasma | 10.0 | 30.34 | 21.64 | 0.71 | 0.97 | 3.18 | 0.68 | 0.27 | 0.04 |
| P013_02 | Plasma | 10.0 | 55.98 | 30.98 | 0.55 | 0.98 | 3.35 | 0.67 | 0.31 | 0.03 |
| P013_03 | Plasma | 10.0 | 47.01 | 25.76 | 0.55 | 0.97 | 3.09 | 0.66 | 0.25 | 0.06 |
| P013_05 | Plasma | 10.0 | 46.14 | 29.35 | 0.64 | 0.97 | 3.14 | 0.66 | 0.28 | 0.05 |
| P013_06 | Plasma | 5.8 | 60.46 | 28.70 | 0.47 | 0.98 | 3.19 | 0.68 | 0.32 | 0.02 |
| P013_07 | Plasma | 4.0 | 61.72 | 29.72 | 0.48 | 0.98 | 3.09 | 0.69 | 0.33 | 0.02 |
| P013_10 | Plasma | 4.4 | 61.88 | 26.97 | 0.44 | 0.98 | 3.16 | 0.67 | 0.29 | 0.03 |
| P013_11 | Plasma | 4.2 | 72.80 | 35.09 | 0.48 | 0.98 | 3.43 | 0.67 | 0.33 | 0.02 |
| P013_12 | Plasma | 8.5 | 56.27 | 27.99 | 0.50 | 0.98 | 3.03 | 0.70 | 0.32 | 0.02 |
| P014_01 | Plasma | 4.0 | 52.38 | 21.95 | 0.42 | 0.98 | 3.02 | 0.65 | 0.27 | 0.03 |
| P015_01 | Plasma | 4.6 | 48.25 | 16.85 | 0.35 | 0.98 | 3.10 | 0.60 | 0.24 | 0.01 |
| P019_01 | Plasma | 5.6 | 59.18 | 31.83 | 0.54 | 0.99 | 2.76 | 0.76 | 0.37 | 0.03 |
| P019_02 | Plasma | 2.7 | 46.70 | 17.22 | 0.37 | 0.98 | 2.95 | 0.62 | 0.23 | 0.03 |
| P020_01 | Plasma | 5.3 | 58.47 | 25.39 | 0.43 | 0.99 | 2.99 | 0.68 | 0.32 | 0.02 |
| P020_02 | Plasma | 5.1 | 56.25 | 26.64 | 0.47 | 0.98 | 3.09 | 0.68 | 0.31 | 0.02 |
| P021_02 | Plasma | 7.1 | 28.95 | 19.10 | 0.66 | 0.99 | 3.09 | 0.69 | 0.33 | 0.03 |
| P025_01 | Plasma | 4.8 | 54.50 | 27.80 | 0.51 | 0.98 | 2.97 | 0.69 | 0.34 | 0.02 |
| P025_02 | Plasma | 4.7 | 56.86 | 30.66 | 0.54 | 0.98 | 3.06 | 0.70 | 0.35 | 0.02 |
| P025_03 | Plasma | 10.0 | 63.49 | 37.76 | 0.59 | 0.98 | 2.88 | 0.72 | 0.37 | 0.04 |
| P025_04 | Plasma | 10.0 | 56.72 | 32.19 | 0.57 | 0.98 | 3.01 | 0.68 | 0.33 | 0.03 |
| P025_05 | Plasma | 10.0 | 55.30 | 31.89 | 0.58 | 0.98 | 2.94 | 0.72 | 0.36 | 0.03 |
| P025_06 | Plasma | 7.1 | 61.69 | 33.00 | 0.53 | 0.98 | 2.98 | 0.70 | 0.35 | 0.03 |
| P025_07 | Plasma | 10.0 | 60.12 | 32.43 | 0.54 | 0.98 | 2.87 | 0.70 | 0.35 | 0.04 |
| P025_08 | Plasma | 6.3 | 60.59 | 33.03 | 0.55 | 0.98 | 2.99 | 0.72 | 0.34 | 0.03 |
| P027_02 | Plasma | 7.1 | 58.98 | 33.72 | 0.57 | 0.99 | 2.94 | 0.72 | 0.36 | 0.02 |
| P028_01 | Plasma | 4.7 | 31.29 | 20.47 | 0.65 | 0.98 | 3.01 | 0.69 | 0.33 | 0.02 |
| P028_02 | Plasma | 10.0 | 54.28 | 32.53 | 0.60 | 0.98 | 3.02 | 0.70 | 0.35 | 0.02 |
| P028_03 | Plasma | 6.6 | 50.58 | 30.21 | 0.60 | 0.98 | 3.15 | 0.69 | 0.32 | 0.02 |
| P028_04 | Plasma | 8.7 | 53.75 | 32.90 | 0.61 | 0.98 | 3.00 | 0.71 | 0.36 | 0.02 |
| P028_05 | Plasma | 5.9 | 58.63 | 30.71 | 0.52 | 0.98 | 3.16 | 0.68 | 0.33 | 0.02 |
| P028_06 | Plasma | 10.0 | 67.92 | 41.19 | 0.61 | 0.99 | 2.98 | 0.73 | 0.39 | 0.03 |
| P028_07 | Plasma | 10.0 | 54.37 | 34.07 | 0.63 | 0.98 | 2.99 | 0.72 | 0.35 | 0.03 |
| P028_08 | Plasma | 10.0 | 48.70 | 23.06 | 0.47 | 0.98 | 3.11 | 0.65 | 0.28 | 0.02 |
| P028_09 | Plasma | 2.9 | 49.72 | 20.79 | 0.42 | 0.98 | 3.28 | 0.62 | 0.24 | 0.02 |
| P028_10 | Plasma | 3.1 | 54.83 | 22.74 | 0.41 | 0.98 | 3.18 | 0.64 | 0.27 | 0.02 |
| P028_11 | Plasma | 10.0 | 55.09 | 28.00 | 0.51 | 0.98 | 3.11 | 0.68 | 0.31 | 0.02 |
| P028_12 | Plasma | 10.0 | 41.12 | 25.20 | 0.61 | 0.98 | 3.04 | 0.68 | 0.29 | 0.02 |
| P028_13 | Plasma | 10.0 | 48.02 | 22.09 | 0.46 | 0.97 | 2.78 | 0.70 | 0.23 | 0.08 |
| P028_14 | Plasma | 10.0 | 51.02 | 32.83 | 0.64 | 0.98 | 3.00 | 0.72 | 0.34 | 0.03 |
| P031_01 | Plasma | 4.1 | 49.36 | 20.70 | 0.42 | 0.98 | 3.14 | 0.62 | 0.25 | 0.02 |
| P032_01 | Plasma | 4.6 | 51.54 | 27.59 | 0.54 | 0.98 | 2.63 | 0.77 | 0.26 | 0.11 |
| P037_01 | Plasma | 4.5 | 29.76 | 19.82 | 0.67 | 0.98 | 3.26 | 0.63 | 0.27 | 0.02 |
| P044_02 | Plasma | 10.0 | 29.28 | 20.44 | 0.70 | 0.98 | 2.83 | 0.73 | 0.31 | 0.06 |
| P044_03 | Plasma | 4.0 | 65.06 | 25.62 | 0.39 | 0.98 | 3.06 | 0.68 | 0.27 | 0.04 |
| P044_04 | Plasma | 10.0 | 70.33 | 40.44 | 0.57 | 0.98 | 2.93 | 0.75 | 0.36 | 0.05 |
| P044_05 | Plasma | 10.0 | 69.19 | 40.23 | 0.58 | 0.98 | 3.06 | 0.73 | 0.35 | 0.04 |
| P044_06 | Plasma | 10.0 | 50.69 | 29.90 | 0.59 | 0.98 | 3.01 | 0.70 | 0.32 | 0.03 |
| P044_07 | Plasma | 10.0 | 56.99 | 35.07 | 0.62 | 0.98 | 3.05 | 0.71 | 0.33 | 0.04 |
| P046_01 | Plasma | 10.0 | 62.52 | 36.20 | 0.58 | 0.98 | 3.17 | 0.66 | 0.32 | 0.04 |
| P010_02 | Plasma | 6.3 | 62.09 | 34.96 | 0.56 | 0.99 | 3.10 | 0.70 | 0.36 | 0.02 |
| C001_01 | Plasma | 4.3 | 48.65 | 15.72 | 0.32 | 0.98 | 3.21 | 0.58 | 0.22 | 0.02 |
| C002_01 | Plasma | 5.9 | 59.98 | 29.17 | 0.49 | 0.99 | 3.13 | 0.68 | 0.33 | 0.02 |
| C003_01 | Plasma | 2.9 | 45.59 | 17.63 | 0.39 | 0.98 | 3.05 | 0.62 | 0.24 | 0.02 |
| C004_01 | Plasma | 3.7 | 50.77 | 22.77 | 0.45 | 0.98 | 3.15 | 0.64 | 0.28 | 0.03 |
| C006_01 | Plasma | 6.7 | 55.12 | 27.61 | 0.50 | 0.98 | 3.07 | 0.68 | 0.31 | 0.02 |
| C007_01 | Plasma | 4.0 | 51.82 | 15.39 | 0.30 | 0.99 | 3.20 | 0.57 | 0.21 | 0.02 |
| C008_01 | Plasma | 2.4 | 49.19 | 16.46 | 0.33 | 0.98 | 3.37 | 0.56 | 0.21 | 0.02 |
| C009_01 | Plasma | 3.8 | 49.04 | 17.92 | 0.37 | 0.98 | 3.17 | 0.60 | 0.25 | 0.02 |
| C010_01 | Plasma | 6.2 | 52.74 | 22.92 | 0.43 | 0.98 | 3.26 | 0.61 | 0.26 | 0.03 |
| C011_01 | Plasma | 5.0 | 60.08 | 26.97 | 0.45 | 0.99 | 3.12 | 0.67 | 0.31 | 0.02 |
| C012_01 | Plasma | 6.6 | 52.05 | 22.91 | 0.44 | 0.98 | 3.08 | 0.66 | 0.25 | 0.05 |
| C013_01 | Plasma | 4.1 | 46.08 | 14.02 | 0.30 | 0.98 | 2.97 | 0.59 | 0.20 | 0.04 |
| C014_01 | Plasma | 6.5 | 66.59 | 32.32 | 0.49 | 0.98 | 3.02 | 0.70 | 0.33 | 0.04 |
| P005_01 | Tissue | 250.0 | 31.01 | 21.85 | 0.70 | 0.99 | 2.79 | 0.72 | 0.31 | 0.03 |
| P013_01 | Tissue | 250.0 | 30.34 | 21.64 | 0.71 | 0.99 | 2.96 | 0.69 | 0.31 | 0.02 |
| P018_02 | Tissue | 250.0 | 31.16 | 23.12 | 0.74 | 0.98 | 2.89 | 0.65 | 0.29 | 0.02 |
| P021_02 | Tissue | 250.0 | 28.95 | 19.10 | 0.66 | 0.99 | 2.80 | 0.67 | 0.28 | 0.02 |
| P028_01 | Tissue | 250.0 | 31.29 | 20.47 | 0.65 | 0.99 | 2.81 | 0.67 | 0.28 | 0.03 |
| P037_01 | Tissue | 250.0 | 29.76 | 19.82 | 0.67 | 0.99 | 2.70 | 0.68 | 0.27 | 0.04 |
| P044_02 | Tissue | 250.0 | 29.28 | 20.44 | 0.70 | 0.99 | 2.78 | 0.67 | 0.29 | 0.03 |
| P048_01 | Tissue | 250.0 | 32.06 | 22.61 | 0.71 | 0.99 | 2.92 | 0.68 | 0.30 | 0.02 |
| P056_01 | Tissue | 250.0 | 29.37 | 19.77 | 0.67 | 0.99 | 2.81 | 0.67 | 0.27 | 0.04 |
| P057_01 | Tissue | 250.0 | 31.00 | 20.80 | 0.67 | 0.99 | 2.95 | 0.66 | 0.29 | 0.02 |
| P063_01 | Tissue | 250.0 | 29.94 | 20.59 | 0.69 | 0.99 | 2.67 | 0.70 | 0.28 | 0.04 |
| P068_01 | Tissue | 250.0 | 30.85 | 19.98 | 0.65 | 0.99 | 2.87 | 0.66 | 0.28 | 0.02 |
| P082_01 | Tissue | 250.0 | 31.32 | 20.02 | 0.64 | 0.99 | 2.83 | 0.66 | 0.28 | 0.02 |
| P083_01 | Tissue | 250.0 | 31.08 | 20.88 | 0.67 | 0.99 | 2.82 | 0.67 | 0.30 | 0.02 |
| P084_01 | Tissue | 250.0 | 30.12 | 21.33 | 0.71 | 0.99 | 2.73 | 0.68 | 0.29 | 0.02 |
| C015_01 | Tissue | 250.0 | 30.44 | 19.73 | 0.65 | 0.99 | 2.68 | 0.68 | 0.24 | 0.06 |
| C016_01 | Tissue | 250.0 | 31.24 | 20.61 | 0.66 | 0.99 | 2.82 | 0.67 | 0.28 | 0.03 |
| C017_01 | Tissue | 250.0 | 32.38 | 21.87 | 0.68 | 0.99 | 2.34 | 0.75 | 0.25 | 0.10 |
| C018_01 | Tissue | 250.0 | 30.03 | 20.61 | 0.69 | 0.99 | 2.40 | 0.74 | 0.25 | 0.08 |
| C019_01 | Tissue | 250.0 | 36.23 | 23.82 | 0.66 | 0.99 | 2.67 | 0.70 | 0.31 | 0.03 |
| C020_01 | Tissue | 250.0 | 30.46 | 20.83 | 0.68 | 0.99 | 2.65 | 0.68 | 0.29 | 0.03 |
| C021_01 | Tissue | 250.0 | 30.90 | 19.96 | 0.65 | 0.99 | 2.70 | 0.69 | 0.27 | 0.05 |
| C022_01 | Tissue | 250.0 | 32.64 | 22.39 | 0.69 | 0.99 | 2.72 | 0.71 | 0.26 | 0.07 |
| C023_01 | Tissue | 250.0 | 30.59 | 20.37 | 0.67 | 0.99 | 2.81 | 0.68 | 0.27 | 0.04 |
| C024_01 | Tissue | 250.0 | 30.90 | 20.46 | 0.66 | 0.99 | 2.79 | 0.71 | 0.28 | 0.03 |

###

# Supplementary figures


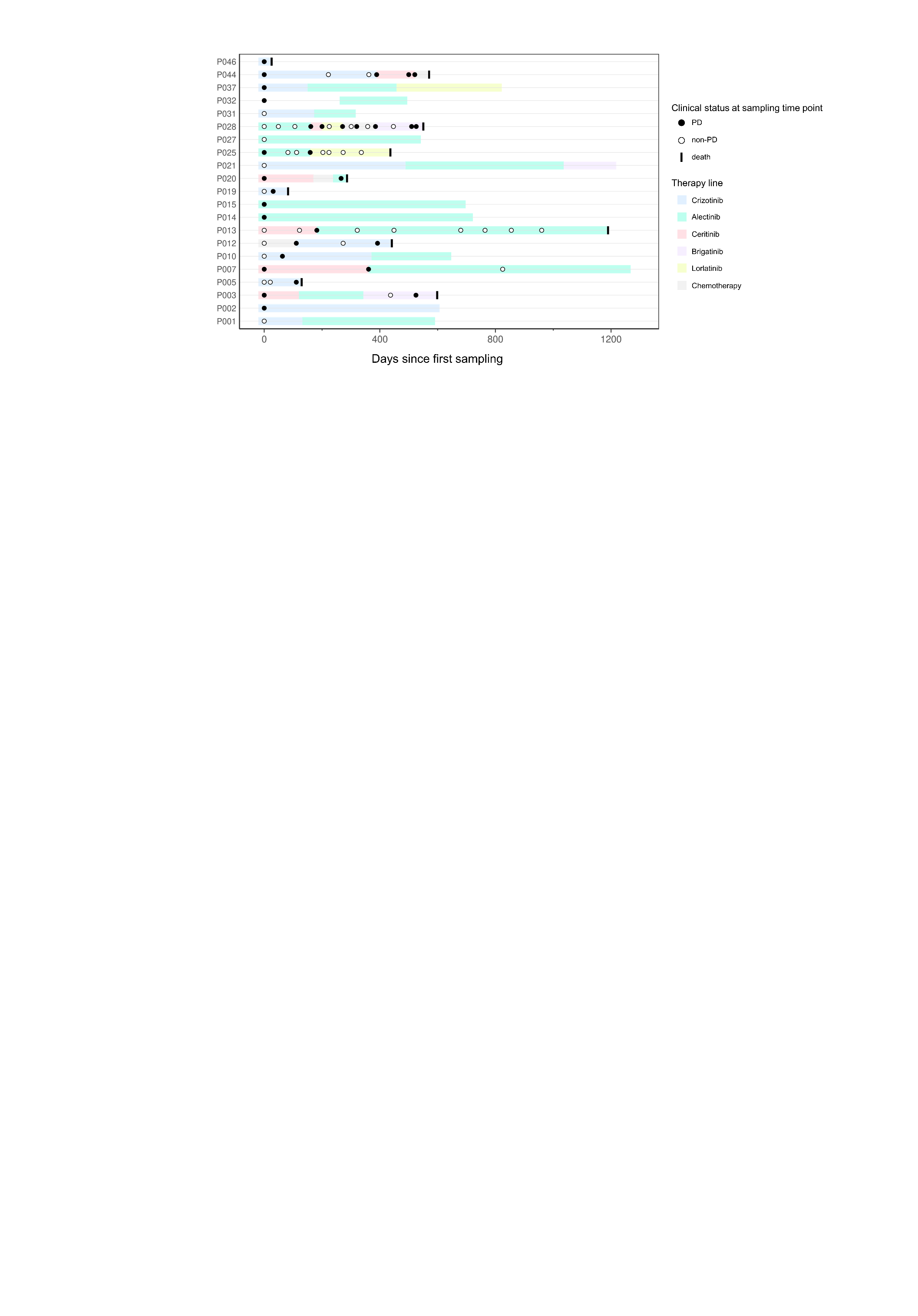


**Figure S1: Timeline of plasma collection and administered therapy regimens.**

###

**
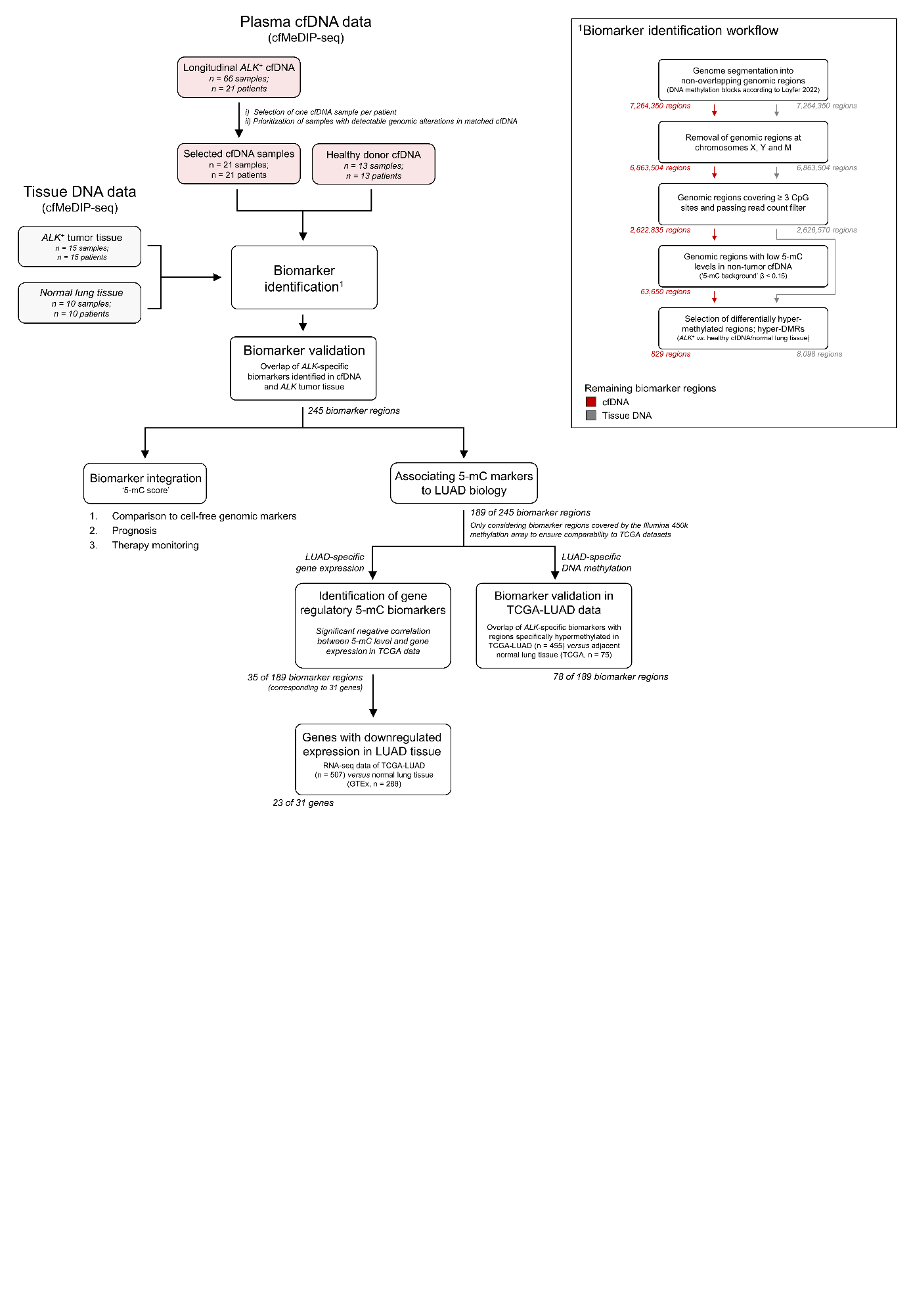
**

**Figure S2: Study flowchart.**

ALK, anaplastic lymphoma kinase; cfDNA, cell-free DNA; cfMeDIP-seq, cell-free methylation DNA immunoprecipitation sequencing; GTEx, The Genotype-Tissue Expression project; LUAD, lung adenocarcinoma; TCGA, The Cancer Genome Atlas.

**###**

**
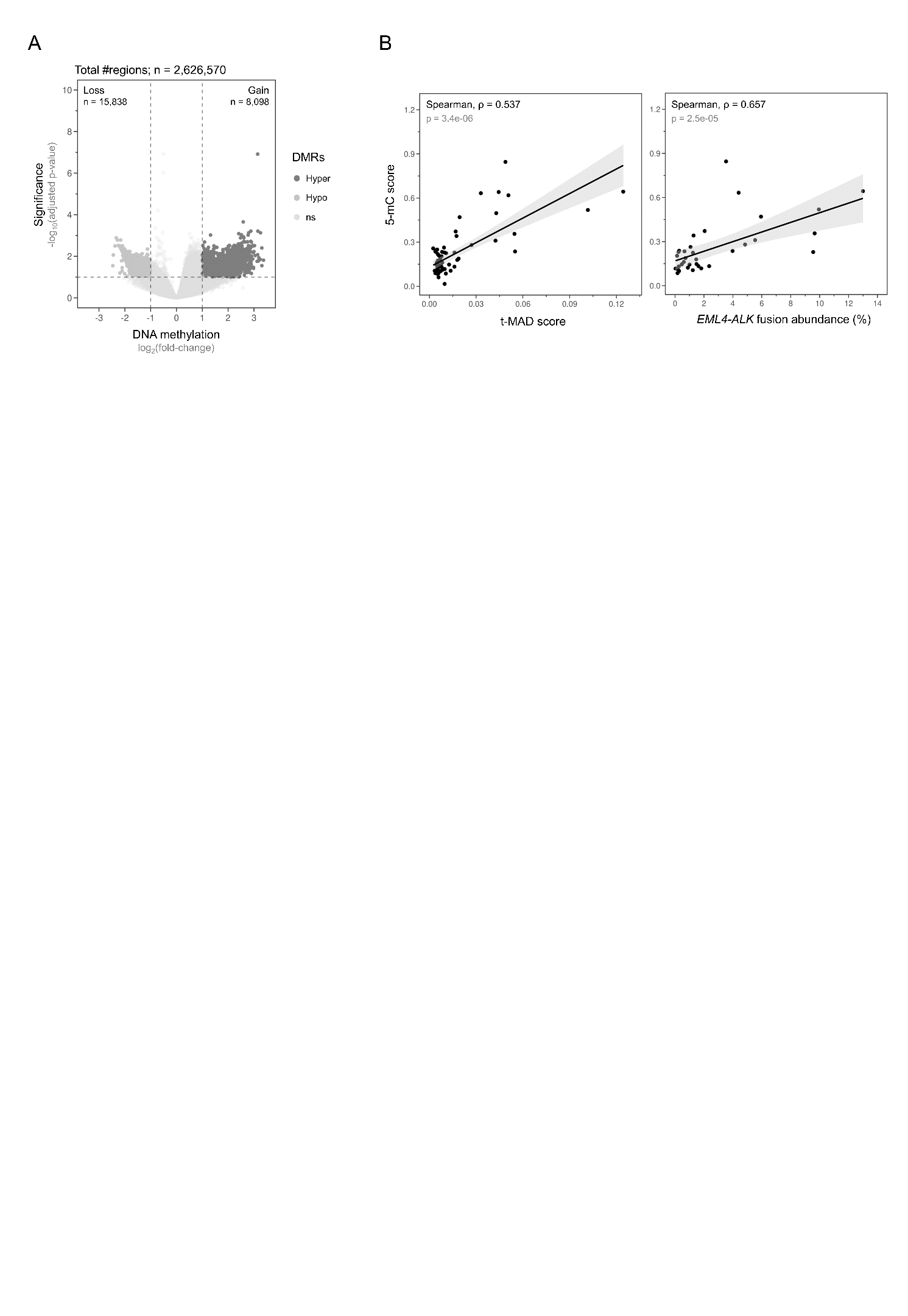
**

**Figure S3: 5-mC biomarker identification and validation.**

1. Volcano plot illustrating significantly hyper- and hypomethylated regions comparing *ALK*-positive tumor to normal lung tissue (without ‘5-mC background’ exclusion).
2. Correlation of 5-mC scores *versus* t-MAD scores (left) and 5-mC scores *versus* *EML4-ALK* fusion abundances (right). 5-mC scores were calculated based on 829 hyper-DMRs identified without stratification for sites concordantly hypermethylated in *ALK* tumor tissue.

*ALK*, anaplastic lymphoma kinase; *EML4*, echinoderm microtubule-associated protein-like 4; DMR, differentially methylated regions; ns, not significant.

###


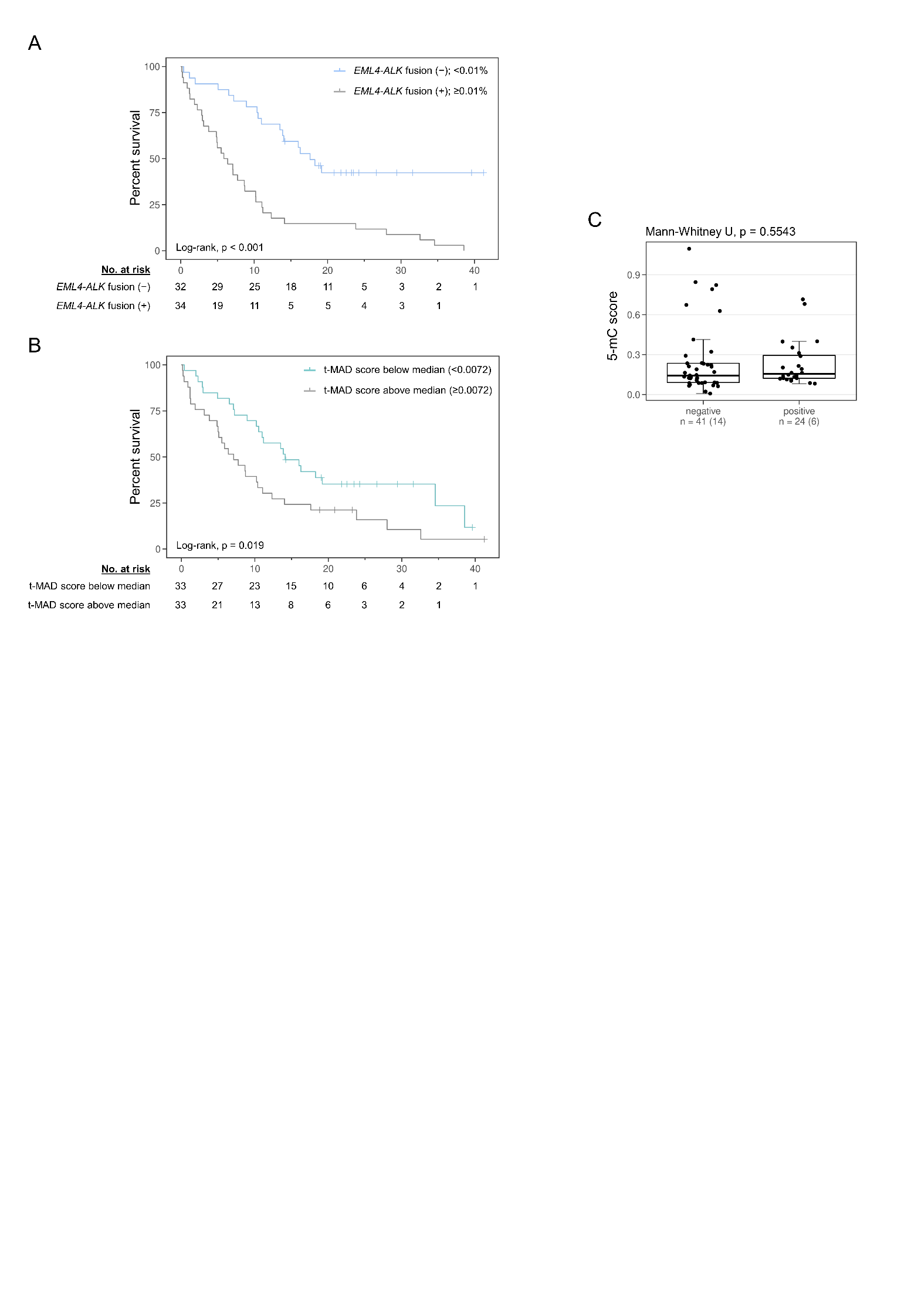


**Figure S4: Association of 5-mC scores to overall survival and molecular risk factors.**

(A-B) Overall survival of *ALK*-positive NSCLC from the time point of plasma sampling according to (A) the detectability of the *EML4-ALK* fusion gene from cfDNA (≥ 0.01%) and (B) the median t-MAD score of all patient samples (0.0072).

(C) 5-mC scores in samples from patients with (positive) and without (negative) *TP53* mutations detected from baseline tissue biopsies. Each dot represents one plasma sample and number of samples and patients (in brackets) are given per group. Box plot center lines indicate the median and boxes illustrate the interquartile range with Tukey whiskers.

*ALK*, anaplastic lymphoma kinase; *EML4*, echinoderm microtubule-associated protein-like 4; *TP53*, tumor protein 53; t-MAD, trimmed median absolute deviation from copy number neutrality.

###


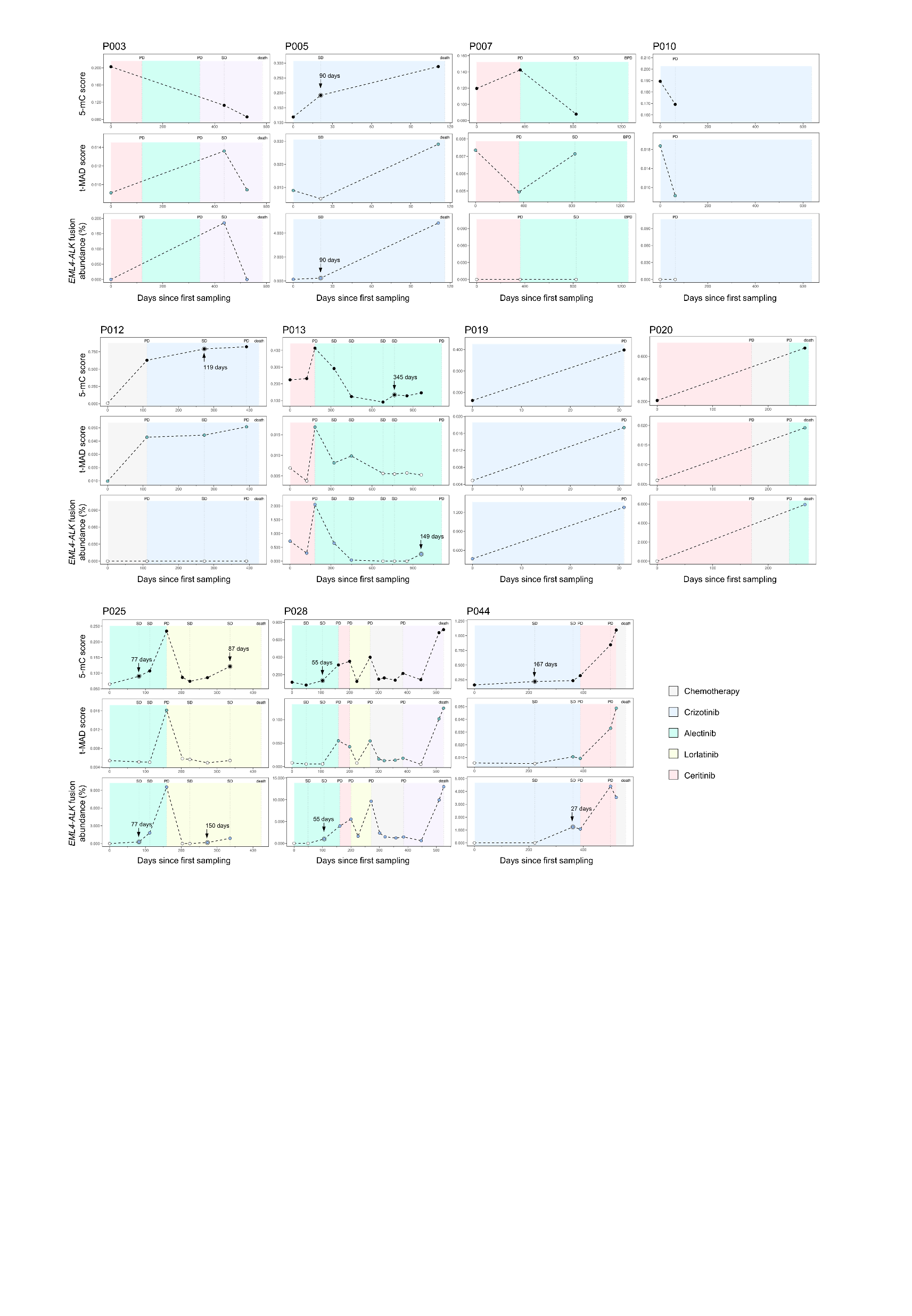


**Figure S5: Cell-free biomarker kinetics of patients with longitudinal plasma collection.**

Longitudinal 5-mC score (top), t-MAD score (middle) and *EML4-ALK* fusion abundances (bottom) of patients with ≥ 2 available plasma samples. Administered therapy regimens are given by the colored backgrounds and radiographic disease assessment, as well as patient death, are indicated above the graph. White dots represent data points below the biomarkers respective limit of detection. Patients with disease progression detectable ahead of radiology are marked with arrows and lead times (in days) are shown.

*ALK*, anaplastic lymphoma kinase; *EML4*, echinoderm microtubule-associated protein-like 4; PD, progressive disease; SD, stable disease; t-MAD, trimmed median absolute deviation from copy number neutrality. ###


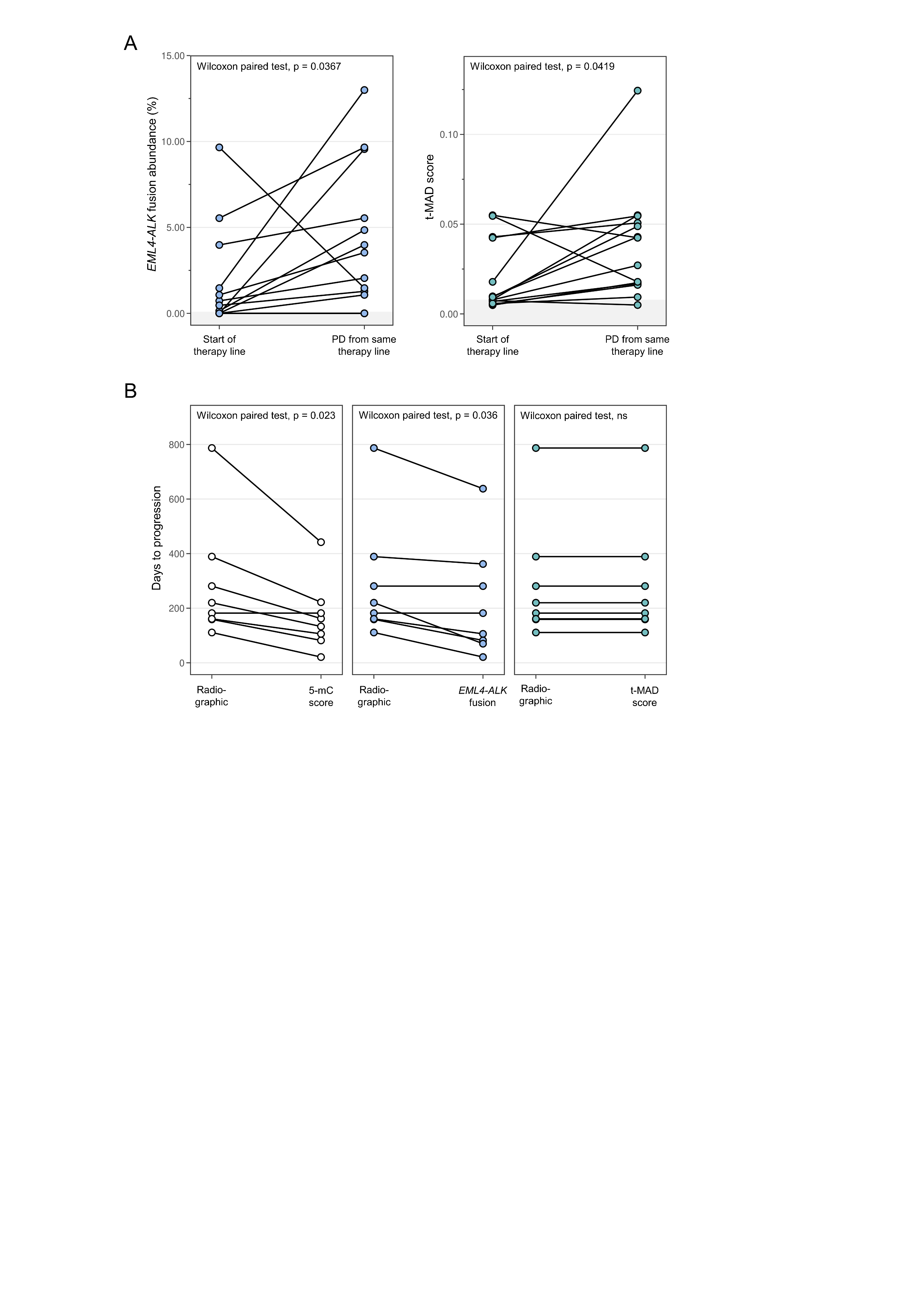


**Figure S6: Indication of disease progression and signs of early molecular progression by 5-mC and genomic biomarkers.**

1. *EML4-ALK* fusion abundance (left) and t-MAD score (right) comparison between samples taken at therapy line start and progression from the same treatment line (n = 13 instances).
2. Difference in days to progression from the start of a therapy line (n = 8 instances), comparing radiologic disease assessment to the 5-mC score (left), *EML4-ALK* fusion abundance (middle) and the t-MAD score (right).

*ALK*, anaplastic lymphoma kinase; *EML4*, echinoderm microtubule-associated protein-like 4; ns, not significant; PD, progressive disease.

###


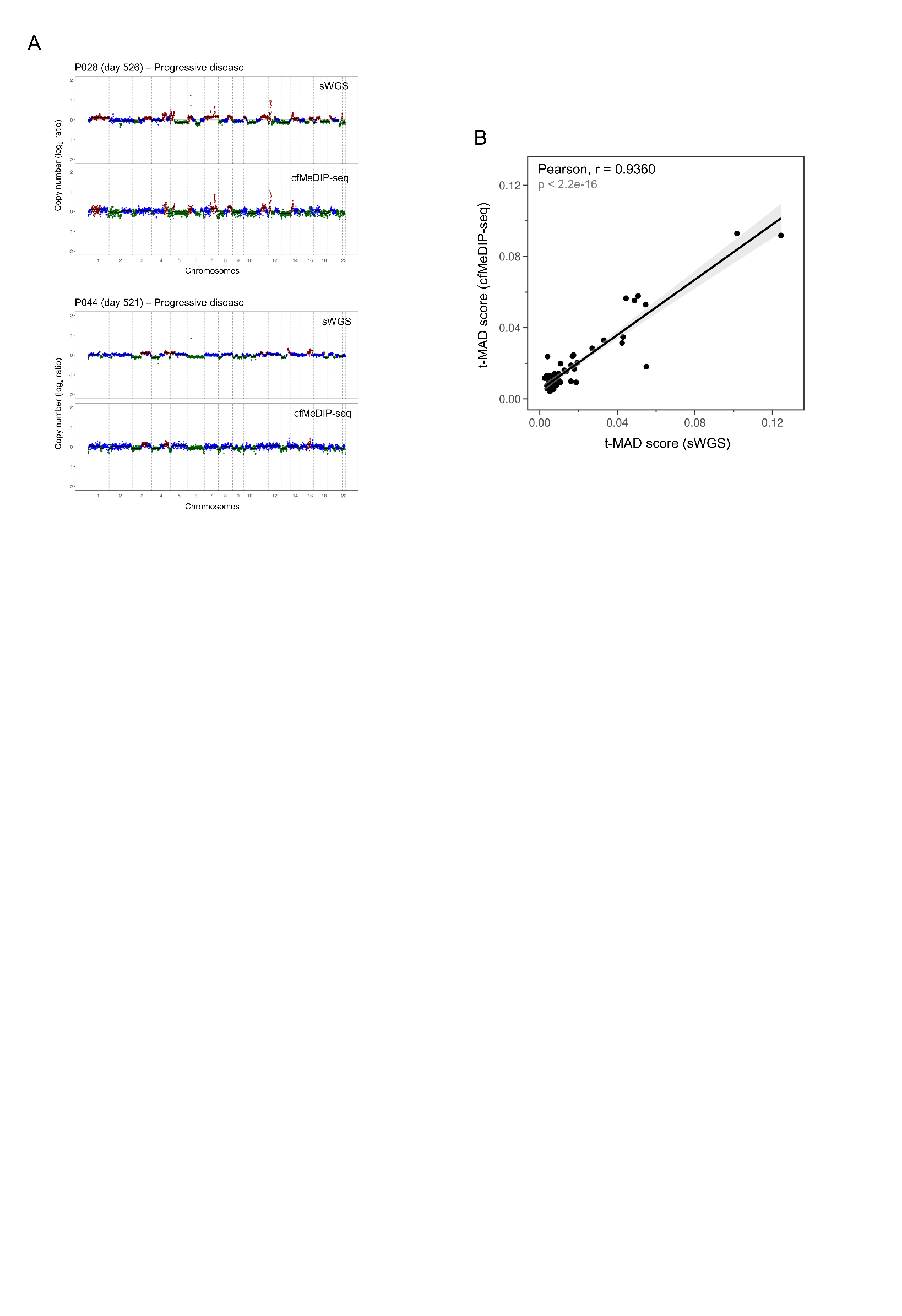


**Figure S7: Comparison of sWGS and cfMeDIP-seq data for the inference of genome-wide chromosomal instability.**

1. Representative copy number profiles of two patient samples taken at disease progression. Profiles were generated from sWGS (top) and cfMeDIP-seq data (bottom). Colors indicate copy number neutrality (blue), deletions (green), copy number gains (brown; 3 copies), and amplifications (> 3 copies).
2. Scatter plot showing the correlation of t-MAD scores determined from sWGS (x-axis) and cfMeDIP-seq data (y-axis).

cfMeDIP-seq, cell-free methylation DNA immunoprecipitation sequencing; sWGS, shallow whole genome sequencing; t-MAD, trimmed median absolute deviation from copy number neutrality.

###
